# Genome-Wide Histone Modifications and CTCF Enrichment Predict Gene Expression in Sheep Macrophages

**DOI:** 10.3389/fgene.2020.612031

**Published:** 2021-01-07

**Authors:** Alisha T. Massa, Michelle R. Mousel, Maria K. Herndon, David R. Herndon, Brenda M. Murdoch, Stephen N. White

**Affiliations:** ^1^Department of Veterinary Microbiology and Pathology, Washington State University, Pullman, WA, United States; ^2^Animal Disease Research Unit, Agricultural Research Service, United States Department of Agriculture, Pullman, WA, United States; ^3^Paul G. Allen School for Global Animal Health, Washington State University, Pullman, WA, United States; ^4^Department of Animal and Veterinary Science, University of Idaho, Moscow, ID, United States; ^5^Center for Reproductive Biology, Washington State University, Pullman, WA, United States

**Keywords:** insulator, promoter, enhancer, innate immunity, alveolar macrophage, sheep, epigenetics, ChIP-seq

## Abstract

Alveolar macrophages function in innate and adaptive immunity, wound healing, and homeostasis in the lungs dependent on tissue-specific gene expression under epigenetic regulation. The functional diversity of tissue resident macrophages, despite their common myeloid lineage, highlights the need to study tissue-specific regulatory elements that control gene expression. Increasing evidence supports the hypothesis that subtle genetic changes alter sheep macrophage response to important production pathogens and zoonoses, for example, viruses like small ruminant lentiviruses and bacteria like *Coxiella burnetii*. Annotation of transcriptional regulatory elements will aid researchers in identifying genetic mutations of immunological consequence. Here we report the first genome-wide survey of regulatory elements in any sheep immune cell, utilizing alveolar macrophages. We assayed histone modifications and CTCF enrichment by chromatin immunoprecipitation with deep sequencing (ChIP-seq) in two sheep to determine *cis-*regulatory DNA elements and chromatin domain boundaries that control immunity-related gene expression. Histone modifications included H3K4me3 (denoting active promoters), H3K27ac (active enhancers), H3K4me1 (primed and distal enhancers), and H3K27me3 (broad silencers). In total, we identified 248,674 reproducible regulatory elements, which allowed assignment of putative biological function in macrophages to 12% of the sheep genome. Data exceeded the FAANG and ENCODE standards of 20 million and 45 million useable fragments for narrow and broad marks, respectively. Active elements showed consensus with RNA-seq data and were predictive of gene expression in alveolar macrophages from the publicly available Sheep Gene Expression Atlas. Silencer elements were not enriched for expressed genes, but rather for repressed developmental genes. CTCF enrichment enabled identification of 11,000 chromatin domains with mean size of 258 kb. To our knowledge, this is the first report to use immunoprecipitated CTCF to determine putative topological domains in sheep immune cells. Furthermore, these data will empower phenotype-associated mutation discovery since most causal variants are within regulatory elements.

## Introduction

Increasingly, livestock researchers are identifying functional variants outside of genes as associated with valuable production traits, supporting the need to molecularly annotate regulatory elements (Ibeagha-Awemu and Zhao, 2015; Zhao et al., 2015; Wang et al., 2017). DNA regulatory elements are sequences associated with a reproducible biological function that can control gene expression through epigenetic modifications (Birney et al., 2007). Human studies consistently document the importance of variants within CRE sequences to critical phenotypic traits. Several groups estimated that over 90% of causal mutations that explain phenotypic variation laid outside of genes within regulatory elements (Hindorff et al., 2009; Maurano et al., 2012; Albert and Kruglyak, 2015). Currently, little is known regarding *in vivo* tissue annotation of regulatory elements in livestock species (Villar et al., 2015; Zhao et al., 2015; Wang et al., 2017, 2018; Naval-Sanchez et al., 2018; Nguyen et al., 2018; Fang et al., 2019; Hall et al., 2020; Kingsley et al., 2020). Therefore, the FAANG consortium recognized this need and formed a global network of researchers for epigenetic discovery in food animal species (Andersson et al., 2015; Tuggle et al., 2016; Giuffra and Tuggle, 2019).

Types of *cis*-acting, DNA regulatory elements (CREs) that control gene expression include active promoters and enhancers, primed enhancers, silencers, and insulators (Dunham et al., 2012). Although CTCF has also been associated with *trans*-acting regulation (Handoko et al., 2011). Promoters are stretches of DNA located at the TSS of genes and serve as scaffolding for promotion, assembly, and initiation of transcription (Birney et al., 2007). Enhancers act more distally and in an orientation independent fashion to activate gene transcription (Banerji et al., 1981). However, enhancers classically must be within the same three-dimensional chromatin domain as their target gene (Schaffner, 2015). Chromatin immunoprecipitation and sequencing (ChIP-seq) (Barski et al., 2007; Johnson et al., 2007) of the post-translational modification marks histone 3 lysine 27 acetylation (H3K27ac) and histone 3 lysine 4 trimethylation (H3K4me3) allowed genome wide identification of active enhancers and active promoters, as demonstrated in early ChIP-seq assays (Heintzman et al., 2007; Won et al., 2008). In addition, H3K27ac often overlapped H3K4me3 regions in active promoters of highly expressed genes (Wang et al., 2008). Potential enhancers that are epigenetically primed but not fully active are marked by histone 3 lysine 4 monomethylation (H3K4me1) alone (Heintzman et al., 2007). H3K4me1, in conjunction with H3K27ac, is found at active distal enhancers (Jambhekar et al., 2019). Lastly, histone 3 lysine 27 trimethylation (H3K27me3) marks broad regions that are transcriptionally repressed or silenced (O’Geen et al., 2011) as the modification is established by the activity of polycomb complexes that help to supercoil the heterochromatin (Dunham et al., 2012; Aranda et al., 2015). Uniquely, some regions are marked simultaneously by methylation at H3K4 and H3K27 termed bivalent regulatory chromatin. Bivalent histone modifications (the combination of H3K4me3 and H3K27me3) were reported to responsively shift gene expression from a poised or primed state to active transcription, most widely studied in embryonic stem cells (Vastenhouw and Schier, 2012). Tissue resident macrophages share features with embryonic stem cells in that they retain the ability to replenish local cell populations (Sieweke and Allen, 2013).

Since regulatory element functions are dependent on three-dimensional chromatin structure within the nucleus, we also sought to define the boundaries of chromatin loops. Chromosomes are compartmentalized into physically interacting segments called TADs (Dixon et al., 2012; Nora et al., 2012) also known as chromatin loops (Rao et al., 2014; Bonev and Cavalli, 2016) that have shared function. Chromatin immunoprecipitation of CCCTC-binding factor (CTCF), denotes insulator regions which anchor domain boundaries (Zhou et al., 2010). The function of *cis*-acting regulatory elements, including those marked by H3K4me3, H3K27ac, and H3K4me1, is generally constrained to genes within the same domain. While histone post translational modifications serve as predictive signals of specific types of regulatory elements, and functions are conserved across species, the exact sequence of the regulatory element is generally not well conserved (Birney et al., 2007; Villar et al., 2015). Therefore, experimental determination of regulatory elements within a variety of tissues is necessary to fully understand unique gene regulatory networks within food animal species.

Host regulatory element variation likely plays a significant role in macrophage immune response to infections. Immunity-related gene regulatory variation has potential to affect production efficiency by altering both the global and tissue-specific transcriptome (Rauw, 2012; Lavin et al., 2014). For example, recent work showed that macrophages can develop trained immunity or innate immune memory which provides non-specific enhanced protection after exposure to pathogens. This non-adaptive immunological memory is reversibly retained in the epigenome of macrophages (Saeed et al., 2014). Trained immunity may be dependent on genetic variants in genes separate from those involved in classical immunological memory (van der Heijden et al., 2018). Furthermore, Salavati et al. (2019) found that sheep immune-related tissues including macrophages have moderate to extreme allele-specific expression. Allele specific expression is commonly attributed to *cis*-acting regulatory variation which provides an understandable mechanism for parent-of-origin or tissue-specific gene expression since *cis*-acting regulatory elements are physically linked to a single allele copy. We have chosen to study alveolar macrophages from sheep lungs both for their tissue-specific gene expression and as a representative cell type to identify immunity related regulatory elements.

Macrophages, as part of the immune system, are a core tissue identified by the FAANG consortium for epigenetic studies (Andersson et al., 2015). Macrophages are professional phagocytes that function in cell-mediated innate immunity at interfaces of the body with the environment and in adaptive immunity as professional antigen presenting cells. Alveolar macrophages in the lungs serve as infection surveillance against airborne pathogens. They also participate in homeostasis in their local tissue microenvironment, a function of specialized tissue-resident macrophages in essentially every organ in the body (Lavin et al., 2014). Macrophages can be hijacked by pathogens like *Mycobacterium bovis*, Ovine lentivirus, *Coxiella burnetii*, *Mycoplasma ovipneumoniae*, *Brucella melitensis*, and *Salmonella enterica*, that cause zoonotic and economically important diseases in sheep: tuberculosis, ovine progressive pneumonia, Q-fever, atypical pneumonia (part of respiratory disease complex), brucellosis, and salmonellosis, respectively (Gendelman et al., 1986; Niang, 1992; Niang et al., 1997; Shannon and Heinzen, 2009; Blacklaws, 2012; Hall et al., 2020). Many of these infectious agents are intracellular organisms that can sequester within host macrophages from the full force of the immune system and manipulate antigen processing and presentation. Elucidation of variation within DNA regulatory elements will aid detection of disease resistant animals that reduce infectious burden within flocks. Genetic determination of resistance and susceptibility can be a crucial tool for disease eradication from individual animal, herd health, and One Health perspectives (Sundberg and Schofield, 2009).

Our objectives for this experiment were to develop a catalog of core histone modifications and of CTCF enriched boundaries in sheep macrophages to locate and functionally annotate regulatory elements. Since CREs compose a far greater portion of the genome than protein coding genes (Moore et al., 2020) lack of annotation in the sheep represents a critical knowledge gap. To the authors’ knowledge, this work is the first epigenetic analysis based on ChIP-seq in any sheep immune cell. We chose native ChIP-seq for greater enrichment and reproducibility of signal (David et al., 2017). As a method of validation, we compared genes near discovered regulatory element regions to RNA-seq data in alveolar macrophages from the Sheep Gene Expression Atlas (Clark et al., 2017). These data presented here will serve as functional epigenetic annotation in sheep immune cells to aid future work on phenotypic-associated variation for important food production, fiber, and immunity related traits.

## Materials and Methods

### Alveolar Macrophage Cell Collection

Animals were cared for and handled according to protocols approved by the Institutional Animal Care and Use Committee at Washington State University under Animal Subject Approval Form 4618. Sheep were humanely euthanized with intravenous sodium pentobarbital and lungs were removed firstly during routine postmortem examination by a veterinarian. No gross lesions were detected in the sheep. Alveolar macrophages were collected from the lungs of 2, 1-year-old, clinically healthy, crossbred (Suffolk, Polypay, and Targhee) ewes using methods modified from those previously described (Gendelman et al., 1984; Cordier et al., 1990; Clark et al., 2017). Briefly, bronchoalveolar lavage fluid was collected by serial lavages with sterile DPBS (Mg^2+^ Ca^2+^ free). Cells were isolated from collected lavage fluid by centrifugation (400 × *g* for 10 min) and washed with DPBS at room temperature. Erythrocytes within the pellet were lysed by suspension in sterile water for 30 s. The harvested cells were confirmed to be morphologically consistent with macrophages on cytological evaluation as others have reported (Sheehan et al., 2005). Cells were stained with trypan blue to assess membrane integrity then counted with an automated cytometer (Nexcelom Bioscience, Lawrence, MA, United States). Aliquots of 5 × 10^7^ live macrophages were suspended in cryopreservation medium (CryoStor CS10, BioLife Solutions, Bothell, WA, United States) and slowly frozen to −80°C in isopropyl alcohol baths (Mr. Frosty, Thermo Fisher Scientific, Waltham, MA, United States) for short term storage.

### Chromatin Immunoprecipitation and Sequencing

#### Isolation of Native Chromatin

Native chromatin isolation and immunoprecipitation was modified from methods published previously for tissues (Wagschal et al., 2007; Maunakea et al., 2010; David et al., 2017; Naval-Sanchez et al., 2018). Additional protocol details are included in Supplementary Methods 1 and provided on the FAANG data portal (see Supplementary Methods 1^1^). Cells and buffers were maintained on ice during all steps. Nuclei were isolated from approximately 5 × 10^7^ unfixed, thawed cells firstly by incubation on ice in hypotonic buffer [0.3 M sucrose, 60 mM KCl, 15 mM NaCl, 5 mM MgCl2, 0.1 mM EGTA, 15 mM Tris-HCl, pH 7.5, and HALT protease inhibitor cocktail (Thermo Fisher Scientific)]. Sodium butyrate 5 mM was included to inhibit histone deacetylases during processing. Next, 0.2% IGEPAL CA-630 detergent (Sigma-Aldrich, St. Louis, MO, United StatesA) was added to the suspension with gentle Dounce homogenization using a tight pestle. The nuclei suspension was then carefully layered onto 8 mL of buffer containing 1.2 M sucrose and centrifuged at 4,000 × *g* for 20 min at 4°C. Detergent layers were removed carefully from the nuclei pellet, then the pellet was resuspended in micrococcal nuclease digestion buffer with protease inhibitors. The pellet was briefly vortexed and then 60 Kunitz units of micrococcal nuclease (M0247S, New England Biolabs, Ipswich, MA, United States) was added for 12 min incubation at 37°C to digest the chromatin into mono- and di-nucleosomes. Addition of 20 mM EGTA quenched the digestion reaction and soluble chromatin fragments were recovered in the supernatant by probe-free, cup horn sonication for 2 × 30 s on ice at high power (260 watts). A sample of purified digested chromatin was checked for adequate fragmentation on an agarose gel and on a fragment bioanalyzer (Agilent, Santa Clara, CA, United States) to ensure oligonucleosome fragment lengths within 100–450 base pairs. Average chromatin fragment size was approximately 150 bp in both biological replicates. Chromatin concentration was then measured by fluorescence quantification using the Qubit dsDNA HS kit (Thermo Fisher Scientific).

#### Immunoprecipitation of Chromatin

Input nucleosomal DNA for each ewe were used as negative controls (no addition of antibody or magnetic beads). Chromatin for immunoprecipitation was pre-cleared by incubation with protein G coupled magnetic beads (Dynabeads, Invitrogen, Waltham, MA, United States). Antibodies to the following targets were used for each chromatin immunoprecipitation: five microliters of anti-H3K4me3, anti-H3K27ac, anti-H3K27me3, anti-H3K4me1, and 10 microliters of anti-CTCF (see Supplementary Table 2 for catalog numbers and lots). Antibodies were pre-bound to magnetic beads at 4°C then the antibody-bead complexes were added to the diluted (50 mM NaCl, 50 mM Tris-HCl pH 7.5, and 5 mM EDTA), fragmented chromatin for overnight incubation in one milliliter volumes with rotation at 4°C. Enriched chromatin was harvested by magnetic bead pulldown, washed with increasing salt buffers (75–175 mM NaCl) to remove non-specific chromatin, and DNA was purified with the iPure kit (Diagenode, Liege, Belgium) as per manufacturer’s recommendation, excluding the cross-linking reversal step. Total amount of immunoprecipitated DNA obtained for each sample was determined by Qubit dsDNA HS analysis.

#### Library Preparation and Sequencing

Sequencing libraries were prepared from 7.5 ng of immunoprecipitated or input control DNA using Truseq ChIP Sample Prep kit (Illumina, San Diego, CA, United States) following the manufacturer’s protocol with 15 PCR cycles to minimize duplication bias and size selection of 250–600 bp to include the bulk of immunoprecipitated fragments ligated to adapters. Preparation for multiplexing was accomplished by utilizing indexing adapters included in the kit. ChIP library size was assessed by Fragment Analyzer (Advanced Analytical Technologies, Ankeny, IA, United States) with the High Sensitivity NGS Fragment Analysis Kit (Agilent, Ankeny, IA, United States), and library concentration was determined by StepOnePlus Real-Time PCR System (Thermo Fisher Scientific) with the KAPA Library Quantification Kit (Kapa Biosystems, Wilmington, MA, United States). Each library was diluted to 4 nM with RSB (10 mM Tris-HCl, pH 8.5), followed by denaturation with 0.1 M NaOH, and 20 pM was clustered in a high-output flow cell using HiSeq Cluster Kit v4 on a cBot (Illumina). After cluster generation, the flow cell was loaded onto HiSeq 2500 for sequencing using HiSeq SBS kit v4 (Illumina). DNA was sequenced with a read length of 50 bp from a single end generating between 41.96 million and 80.15 million filter-passed reads for each library. These were derived from a total of 644,923,132 reads for the experiment that passed initial sequencing quality filters (97% pass-rate) (Table 1 and Supplementary Data 15).

**TABLE 1:** Summary of read counts from ChIP-seq assays.

ChIP-seq target	Ewe A		Ewe B	
	Total reads	Usable fragments	Total reads	Usable fragments
Input Control	43,513,683	33,901,350	44,568,055	34,245,907
H3K4me3	41,963,508	23,545,621	43,705,079	23,796,392
H3K27ac	46,534,416	33,599,474	46,205,646	34,309,656
H3K27me3	72,339,834	45,918,247	68,038,026	47,130,715
H3K4me1	78,646,889	59,258,883	80,153,553	48,412,817
CTCF	44,138,652	28,139,080	42,575,226	28,717,487

*Total reads include all raw data from sequencing. Usable fragments are defined consistent with ENCODE standards as reads that map to a single best location (quality filter -q 5), with optical duplicates removed as flagged by MACS2. Additional mapping statistics are in the Supplementary Data 15.*

### Analysis of ChIP-Seq Data

Sheep ChIP-seq sequencing files generated for this article are publicly available in the ENA database and FAANG data portal under project accession PRJEB40528 (ERP124181). Optional parameters used for all bioinformatics tools and detailed bioinformatics protocol are included in Supplementary Table 3.

Sequencing data bcl files were converted to fastq format and adaptor sequences were trimmed using bcl2fastq2 (Illumina). Reads were quality checked with FastQC software (Andrews and Babraham, 2016) with attention to duplication rate (Supplementary Data 15). Sequence reads were mapped to the unmasked Rambouillet sheep genome (Oar_rambouillet_v1.0, GCA_002742125.1, Worley, 2017; Salavati et al., 2020), that excludes the mitochondrial genome, with BWA v0.7.17 (Li and Durbin, 2009) (see Supplementary Data 15 and Supplementary Data 4 supplementary results for additional mapping details). Reads were sorted and indexed with Picard v2.9.2^2^. Reads were filtered for quality and unique mapping with SAMtools v1.9 (Li et al., 2009). Peaks for histone modifications and CTCF were found for each animal individually using MACS2 v2.1.1 at FDR cut-offs of less than 5% (Zhang et al., 2008; Feng et al., 2012). Effective genome size of the sheep was specified as 2.62 × 10^9^ bp based on the Golden Path Length from ENSEMBL. The broad peak calling option in MACS2 was enabled to calculate both narrow peaks and broad block binding of the H3K27me3 and H3K4me1 datasets. A third set of peaks were called from pooled reads from both animal replicates to maximize sensitivity; these pooled peaks were subsequently filtered for only those called in both individual animals. Overlap between all three peak sets, each individual animal and the pooled reads, were determined with bedtools v2.26.0 and bedops v2.4.38 to create the reproducible consensus peaks (Quinlan and Hall, 2010; Neph et al., 2012). These reproducible consensus peaks were used for all downstream analysis of regulatory elements. Regulatory elements were categorized into active promoters (H3K4me3-enriched regions, with or without overlapping H3K27ac enrichment), active enhancers (all regions enriched for H3K27ac, and regions with H3K27ac only), primed enhancers (H3K4me1-enriched regions), silencers (H3K27me3-enriched), and insulator chromatin domain boundaries (CTCF-enriched).

The called peaks were annotated with the nearest gene and genomic feature type using the annotatePeaks.pl program in HOMER v4.10.4 (Heinz et al., 2010) and the NCBI *Ovis aries* Refseq Annotation Release 103 (O’Leary et al., 2016) for the Oar_Rambouillet_v1.0 genome (GCF_002742125.1). The definition of genomic promoter features was manually adjusted to regions within 2 kb of any gene TSS in the Annotation Release. GO analysis was completed from ChIP-seq target associated gene lists with PANTHER (Mi et al., 2019). HOMER findMotifs.pl was used to scan consensus peaks for transcription factor protein binding motifs (see Supplementary Methods 1 for further details). Correlation analysis and conversion of BAM files to normalized, input-subtracted bigwig files for visualization was completed with deepTools v3.3.0 (Ramírez et al., 2016) (Supplementary Methods 1).

### Comparison of ChIP-Seq Data to Public RNA-Seq Data

Processed gene expression data in sheep alveolar macrophages from publicly available mRNA-seq datasets were obtained from supplementary files provided by Clark et al. “Supplementary Dataset 1. Gene expression level atlas as TPM (unaveraged)” available at https://doi.org/10.1371/journal.pgen.1006997.s004 (Clark et al., 2017). The authors also provided the processed data available for download through the University of Edinburgh DataShare portal at http://dx.doi.org/10.7488/ds/2112. This processed data was derived from paired end alveolar macrophage transcriptomic RNA-seq from two adult, females: a Texel × Scottish Blackface available at the ENA database under study accession number PRJEB19199^3^ at sample accession SAMEA5535418 run accession ERR2074323 (Clark et al., 2017) and a Texel from study accession PRJEB6169^4^, published previously (Jiang et al., 2014).

The processed RNA-seq data from female alveolar macrophages was filtered by genes expressed equal to or greater than 1.0 TPM in at least one animal. Mitochondrial genes were removed as ChIP-seq data is from nuclear chromatin only. This yielded a list of 12,042 genes expressed in alveolar macrophages from either individual female at TPM ≥ 1. These genes were then ranked by average TPM for comparison to ChIP-seq peak enrichment. Consensus BED files for each histone modification were annotated with ChIP-seq read count per peak region from the pooled mapped reads of both Crossbred ewes, then ranked from highest to lowest by count. Rank of peaks by total read count and their corresponding nearest gene were compared to rank of genes from RNA-seq TPM with Spearman’s Rho correlation test since the data was non-parametric. Unidentified *LOC* open reading frames that were mapped to Oar_v3.1 without gene names and that could not be converted to an open reading frame in Oar_Rambouillet_v1.0 with NCBI Genome Remap were removed before comparisons.

## Results

### Summary of Quality Metrics

Chromatin immunoprecipitation and sequencing for four histone modification marks: H3K4me3, H3K27ac, H3K4me1, H3K27me3, and CTCF were completed on two animal replicates to identify regulatory elements in sheep alveolar macrophages. Negative controls consisted of input fragmented chromatin for each animal sequenced to a similar depth. Mean mapping rate for raw reads was 98.58% to the Rambouillet genome assembly (Oar_rambouillet_v1.0) (see Supplementary Data 15 for detailed mapping rates). Non-duplicated fractions of reads were high between 0.80 and 0.94 indicating good library quality. Usable fragments exceeded 23 million reads for all narrow marks and 45 million reads for broad marks (Table 1). Correlation of mapped filtered reads for all ChIP-seq datasets sorted by each chromatin mark rather than by individual animal (Supplementary Data 15 and Supplementary Figure 5). This confirmed reproducibility of antibody enrichment between the two animal replicates (Pearson’s correlation coefficient: 0.94–0.99) (see Supplementary Figures 5, 6 for additional animal replicate comparisons). Cumulative enrichment “fingerprint” plots showed significant enrichment above the background, particularly for narrow marks such as H3K4me3 (Supplementary Figure 7). NSC and RSC values confirmed significant enrichment in immunoprecipitated datasets compared to input controls, exceeding 1.05 and 2.17, respectively, in all datasets (Supplementary Data 15).

### Regulatory Element Region Characteristics, GC Content, and Genome Coverage

**FIGURE 1:**
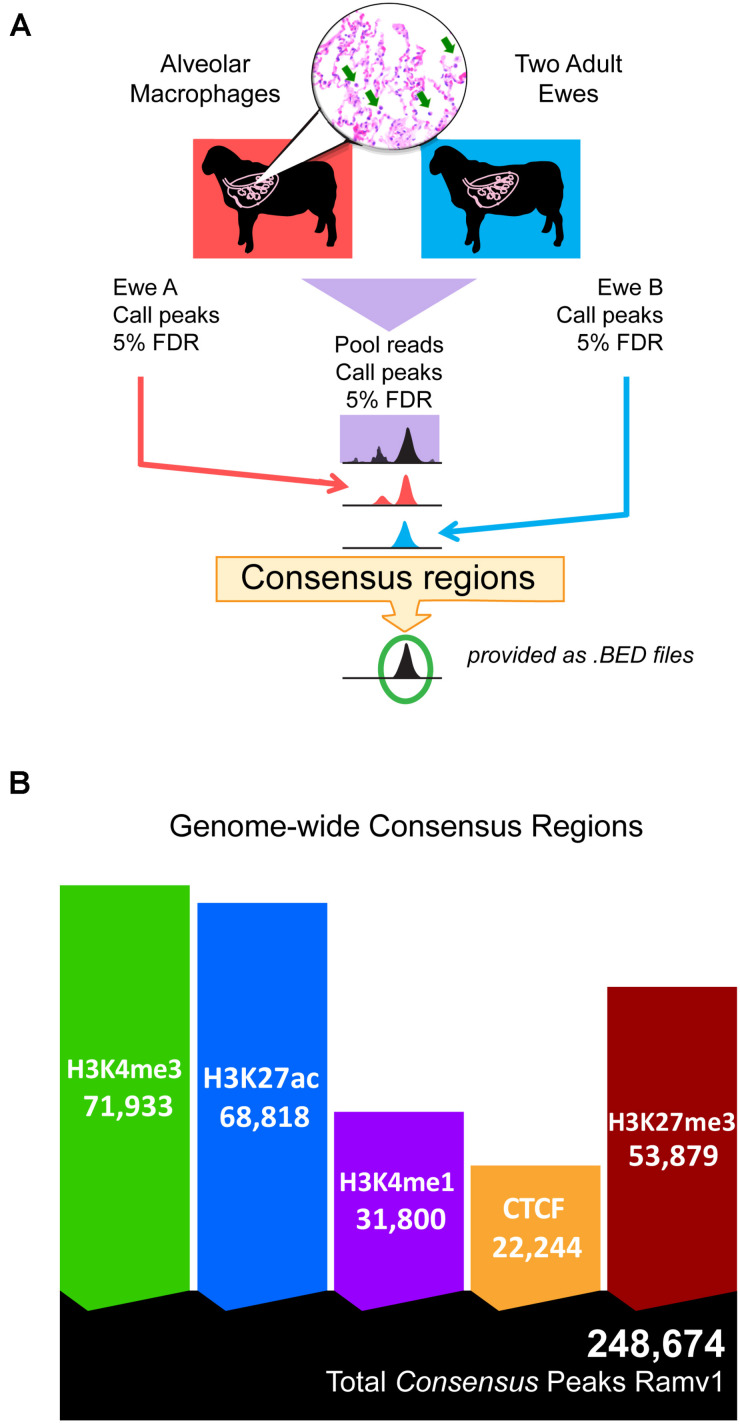
Study design and total consensus regulatory elements identified in alveolar macrophages. **(A)** Schematic overview of the study design for determining consensus regulatory element regions, statistically reproducible with a FDR cut-off of 5% in all three datasets: pooled reads, ewe A, and ewe B. **(B)** Native ChIP-seq yielded many reproducible consensus regions for all chromatin marks. Ramv1 is Oar_rambouillet_v1.0 genome.

Regulatory elements were defined by regions of ChIP-seq signal enrichment along the genome for each of the five chromatin marks; total analysis included ten antibody-enriched, epigenomic datasets from alveolar macrophages. Significant regions were called at 5% FDR in both individual animals and then in pooled reads. In total 491,635 and 446,798 regulatory elements were defined in each individual animal (Supplementary Figure 8 and Supplementary Data S15-Table 3). Together regulatory elements covered between 8.79 and 8.23% of the genome in individual animals. We then filtered the set of significantly enriched regions in the pooled reads to select only those that were also significant in both individual animals (see Figure 1A for study design). We termed these reproducible regions of signal enrichment as consensus regulatory elements. This yielded 248,674 consensus regulatory elements in sheep alveolar macrophages (Figure 1B). Consensus regions were putatively assigned to regulatory element classes. Active *cis*-acting regulatory elements include 71,933 regions marked by H3K4me3 classified as promoters and 68,818 marked by H3K27ac grouped as enhancers or highly active regions. Regions enriched for H3K4me1, considered primed and active enhancers were discovered at 31,800 genomic locations that included both broad and narrow regions of signal enrichment. Silencers were regions with signal enrichment for H3K27me3 found at 53,879 broad regions that cover long stretches of DNA. Lastly, 22,244 very narrow regions marked by CTCF were identified that denoted genomic locations of insulators indicative of chromatin domain boundaries. Consensus regulatory elements were used for further analysis since there was acceptable agreement amongst animal replicates (Supplementary Datas 5, 6, 15).

Altogether, consensus regulatory elements from macrophages cover 11.77% of the sheep genome. Promoter signal enrichment covered 2.77% of the genome and regions were narrow, short stretches of DNA, with a median length of 0.81 kb (Table 2). Promoter regions had higher GC nucleotide content compared with other regulatory elements and the background GC content of the sheep genome (Table 2). Active enhancers (H3K27ac) comprised a slightly longer portion of the genome than promoters. Active enhancer regions also had increased GC nucleotide content compared to the genomic average but less so than promoters. Broad and narrow primed enhancers marked by H3K4me1 covered larger regions of the genome (Table 2) and had neutral to mildly depleted GC content. Insulator regions enriched for CTCF were fewer and narrowest with a median length of 0.76 kb occupying the least percentage of the genome. Silencers marked by H3K27me3, covered the largest portion of the genome at 6.05% and displayed broad blocks of signal enrichment at 2.5 kb median length. Silencer regions were markedly depleted of GC nucleotide content compared with other regulatory elements and the genome background content.

**TABLE 2:** Consensus regulatory element region details.

Chromatin mark	Total genome coverage %	Median region length in bp (Q1–Q3)		Average GC base content %
H3K4me3	2.77	818	(534–1,320)	47.9
H3K27ac	2.85	843	(542–1,399)	43.2
H3K4me1	3.76	2,419	(1,435–4,059)	40.9
CTCF	0.71	764	(534–1,089)	39.4
H3K27me3	6.05	2,488	(1,542–3,962)	38.4

*The Oar_Rambouillet_v1.0 genome assembly was used to calculate coverage and GC content for regions captured by each ChIP-seq mark. The assembly is 2,869.9 megabases (Mb) with an estimated effective genome size of 2,620 Mb and calculated 41.9% GC base content. Q1–Q3 is quartile 1 and quartile 3 for interquartile range.*

### Regulatory Elements Have Combinations of Multiple Chromatin Marks

**FIGURE 2:**
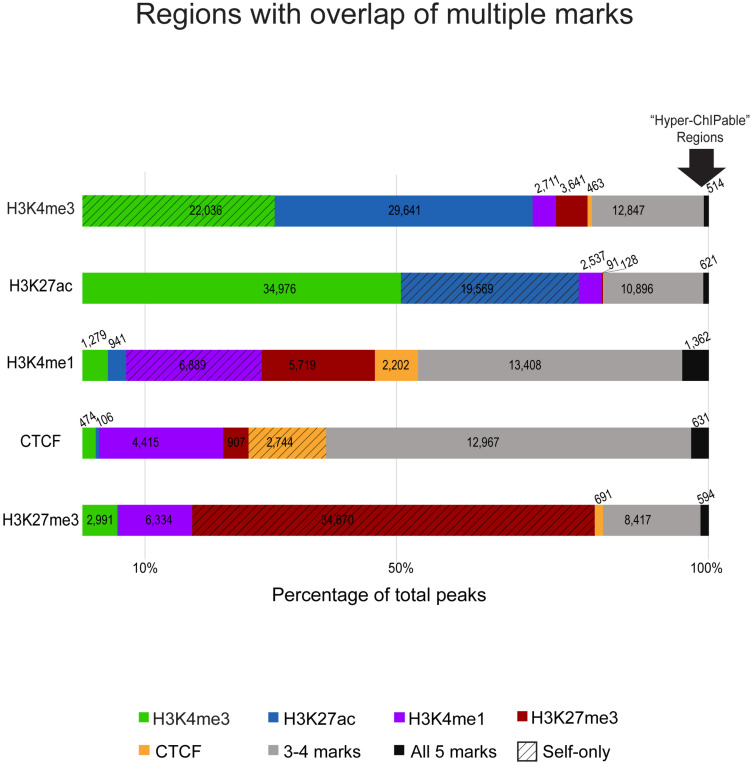
Overlaps of chromatin marks with one another as a percentage of consensus regions for each ChIP-seq target. The segmented bar graphs show regions enriched in each respective ChIP-seq mark color coded by overlap with other marks, the number of peaks from that respective mark that overlap with peaks from other marks are noted in each color-coded segment. Textured bars are regions exclusive to that mark (only marked by self) that did not have enrichment for other marks. Gray bars are regions that have three or four overlapping chromatin marks in that region. On the far-right, the arrow indicates black bars representative of regions with significant signal enrichment in all immunoprecipitated datasets with no specificity for the ChIP-seq antibody target, these were considered putative “hyperChIPable” regions.

Detected regulatory element regions had either a single type of chromatin mark or a combination of enrichment from multiple marks in that stretch of DNA (Figure 2). Most combinations of marks were between those associated with active gene expression (H3K4me3, H3K27ac, and H3K4me1) whereas the repressive mark H3K27me3 had much fewer regions with overlap by another mark. Generally, H3K4me3 and H3K27ac active marks had greater numbers of overlapping regions and H3K27me3 silencer regions had few overlapping regions with either active mark. Boundary regions between these two types of chromatin were often marked by CTCF and H3K4me1 enrichment. Shown in Figure 2, promoters and enhancers captured 54–65% of the same regions. However, not all promoters marked by H3K4me3 appeared to be active as only 40,112 of them were also marked by H3K27ac. In fact, H3K4me3 regions were also occasionally enriched for the repressive mark H3K27me3. Promoter regions that did not have H3K27ac signal were mostly enriched for only H3K4me3 (22,036; 70% self only) and did not have overlap with other chromatin marks.

We also analyzed enhancer associated overlap of marks to elucidate primed, transitional, and active enhancers used in sheep macrophages. Overall, 87,458 putative enhancer regions were identified as those enriched for H3K27ac or H3K4me1, or a combination of both. H3K27ac enrichment was found at 28% of the regions marked by H3K4me1 which yielded 8,932 putative highly active enhancers (Supplementary Data 15). However, we also found that 19,569 putative active enhancers were exclusively marked by H3K27ac (self-only) and were not overlapped by enrichment for H3K4me1 suggestive of regions with a different *cis*-acting regulatory function. Primed enhancers, with H3K4me1 signal enrichment, and CTCF -enriched insulators shared notable overlap since approximately 80% of total CTCF regions were also marked by H3K4me1 (Supplementary Data 15). Both CTCF and H3K4me1 signal enriched regions had greater overlap by multiple marks (42–58%, Figure 2 gray bars) than in other immunoprecipitated datasets.

Most silencer regions, 64%, are only enriched for H3K27me3 signal consistent with the expected prediction of heterochromatin that would exclude the other ChIP-seq targets we assayed (Figure 2, red hashed bar). Approximately 15% of silencer regions have some overlap with H3K4me1 indicative of primed or transitional regulatory elements at boundary regions. Acetylation and trimethylation of H3K27 are essentially never found in the same regions (0.1%, Supplementary Data 15), except where regions were enriched in most or all immunoprecipitated datasets. Interestingly, 1520 genomic regions had significant signal enrichment in all histone modification and CTCF datasets (Figure 2, black bars).

### Regulatory Element Annotations and Genomic Localization

**FIGURE 3:**
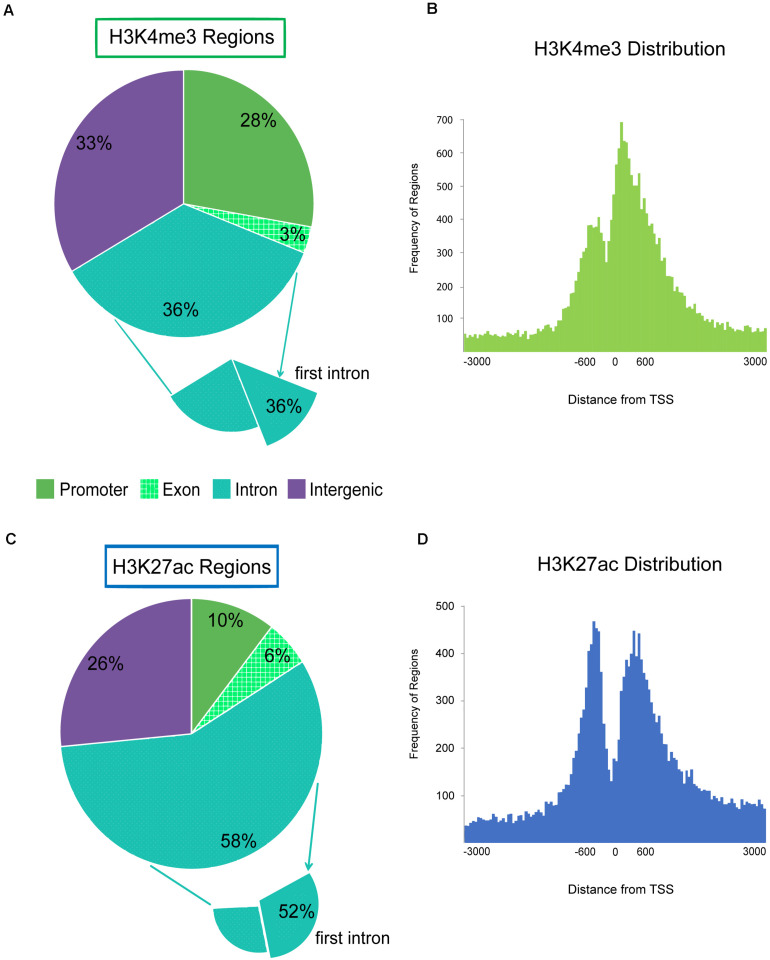
Genomic localization of active promoters and enhancers, H3K4me3 and H3K27ac. Pie charts display the genomic feature (promoter, exon, intron, or intergenic) that regions from each active ChIP-seq target fell within. Promoter is defined as features that fall within 2 kb of the 5′ end of genes. Histograms display the distribution of ChIP-seq regions around the transcription start site (TSS) 0 at the 5′ end of genes by distance in bp. ChIP-seq regions were binned into number per 50 bp segment. **(A)** Genomic locations of H3K4me3 enriched regions by feature. The subset that were found within the first intron is also shown. **(B)** Distribution of H3K4me3 regions around the TSS of genes. **(C)** Genomic locations of H3K27ac enriched regions by feature; and **(D)** H3K27ac regions around gene TSS.

Each regulatory region was binned into a genomic category (promoter, intron, exon, or intergenic) and annotated with the nearest Refseq gene. The majority of H3K4me3 enriched regions were located within genes (intron and exon) or near the 5′ end of genes within 2 kb of TSS annotated as promoter regions (Figure 3A and Supplementary Figure 9). Twenty-eight percent of all H3K4me3 enriched regions were within 2 kb of the annotated TSSs of genes and pseudogenes. Nearly half (49%) of the regions distal to the TSS were within the first intron or first exon of genes. The pattern of H3K4me3 signal around gene TSSs was bimodal with high enrichment of promoters regions 500 base pairs upstream of the gene and a maximal enrichment at 200 base pairs downstream of the TSS with severe depletion of signal at the TSS (Figure 3B). Inspection of H3K4me3 regions that were within 2 kb of an annotated TSS, revealed 11 were associated with miRNAs in sheep and 295 were associated with tRNA. Overall, 3.6% of active enhancer and active promoter regions were associated with tRNA genes.

More than half of H3K27ac enhancer regions were annotated within introns (Figure 3C). Half of those regions were within the first intron which indicated enrichment for active enhancers near the 5′ end of genes. Active enhancer signal had a similar bimodal distribution around the TSS of genes as promoters, but the maximal signal was located approximately 450 bp upstream of genes, with a second peak of signal approximately 450 bp downstream of the TSS (Figure 3D). However, on average, H3K27ac-exclusively enriched enhancer regions were more distal, 36 kb from the nearest TSS with 21% greater than 50 kb away from the nearest gene. Only 4% of the H3K27ac-exclusive regions were within 2 kb of a TSS. Silencers exclusively marked by H3K27me3 were further from genes than other ChIP-seq targets at an average of 53.5 kb from TSS with 34% greater than 50 kb away.

### Promoters Predicted Actively Expressed Genes From RNA-Seq

**FIGURE 4:**
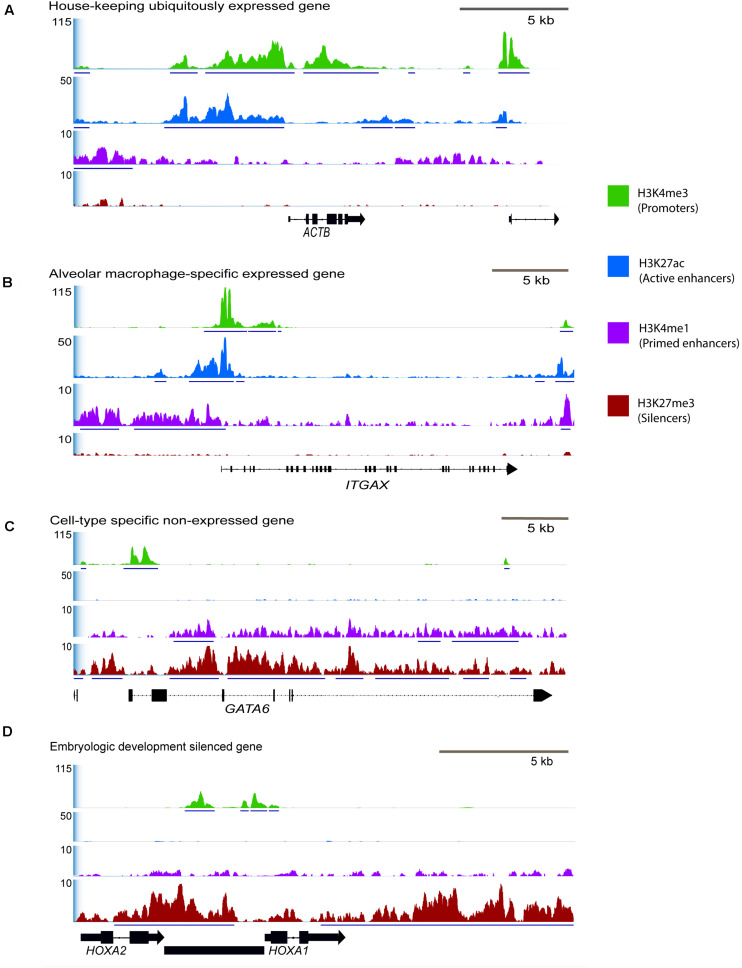
Selected consensus regions of ChIP-seq signal at active and repressed genes in macrophages. Signal enrichment along the *Y*-axis is displayed as average reads per genomic content (RPGC) normalized for sequencing depth to 1× genome coverage. Input control signal was subtracted from the profiles to remove noise. The *X*-axis represents the chromosomal location with size bar given in kb. **(A)** Region displaying a housekeeping gene that we would be constitutively expressed in all cells and have active promoter and enhancer peaks, *ACTB* (actin-beta) from chromosome 24. **(B)** Tissue-specific gene actively expressed in alveolar macrophages, *ITGAX*, chromosome 24, and **(C)** tissue specific gene that is not expressed in alveolar macrophages, *GATA6*, chromosome 23. **(D)** Developmental genes that should be silenced with broad H3K27me3 signal, *HOXA1* and *HOXA2* on chromosome 4 that are not expressed in fully differentiated macrophages. In this region there is also an unannotated gene (predicted lncRNA) that has a bivalent promoter enriched by both H3K27me3 and H3K4me3. See Supplementary Figure 10 for signal around additional immune-related genes.

Exactly half of all genes annotated in the Rambouillet reference (Refseq Annotation release 103) were associated with an H3K4me3 enriched regulatory element. Regulatory elements with H3K4me3 signal were identified with gene-rich regions of the genome, 77% were within 20 kb of the nearest gene. Regions with ChIP enrichment for active promoters and enhancers, H3K4me3, H3K27ac, and H3K4me1 were at constitutively expressed housekeeping genes including *POL3D*, *ACTB* (Figure 4A), and *GAPDH* and many had moderate signal enrichment. Active promoters were also found at macrophage tissue-specific genes like *PPARG* (Supplementary Figure 10) and at environment-specific genes like *ITGAX* that are highly expressed in RNA-seq (Figure 4B). Several tissue-specific highly expressed genes were associated with high ChIP-seq signal (Figure 4B). Lineage-specific genes that are not expressed in alveolar macrophages such as *GATA6* (Figure 4C) had enrichment of H3K4me3 at promoter regions but lacked distal enhancers (H3K4me1 and H3K27ac) and were not enriched for H3K27ac at promoters. Developmental genes which are not expressed in adult alveolar macrophages had broad regions of enrichment for H3K27me3 (Figure 4D).

**FIGURE 5:**
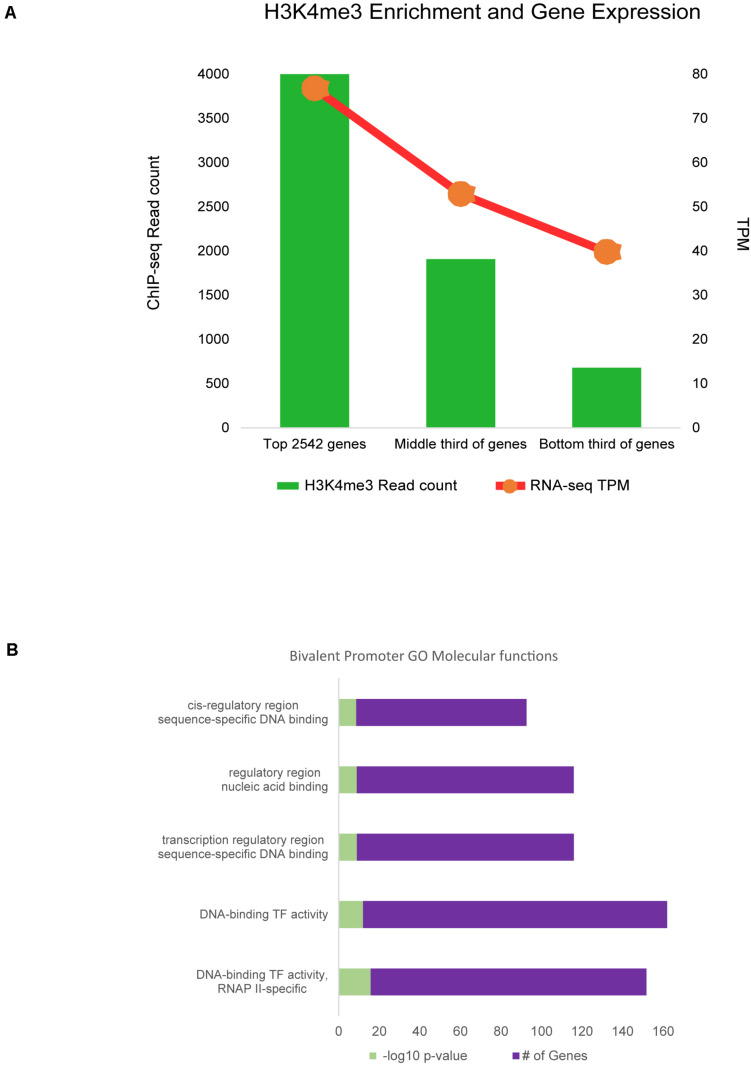
Promoter regulatory elements and gene expression. **(A)** RNA-seq gene expression was associated with signal enrichment of active promoters marked by H3K4me3. RNA expression is shown as transcripts per million (TPM). **(B)** Bivalent promoter (H3K4me3 and H3K27me3) associated GO analysis annotations for categories of molecular functions.

Promoter regions enriched for H3K4me3 were then filtered for those within only 2 kb of annotated genes, as these were most likely to be a correct match between regulatory element and TSS. Approximately 73% of genes with active H3K4me3 enrichment within 2 kb of the TSS were also expressed in RNA-seq data from sheep alveolar macrophages in the Sheep Gene Expression Atlas. Analysis of regions with H3K4me3 and H3K27ac enrichment showed 78% were associated with gene expression from RNA-seq regardless of distance from that annotated gene. Maximum signal enrichment of H3K4me3 at promoter peaks within 2 kb of TSS was positively correlated with gene expression TPM when compared to RNA-seq data (*r* = 0.28, *P* = 10^–130^). The top third H3K4me3 enriched regions corresponded to genes from the RNA-seq data with an average expression of 77 TPM (Figure 5A). Within the bottom third of H3K4me3-enriched regions the average expression of nearest genes was 40 TPM. Signal at promoter regions with enrichment for both H3K4me3 and H3K27ac did not have a quantitatively stronger correlation with gene expression than did H3K4me3 signal alone (*r* = 0.12).

Gene ontology analysis of genes associated with both H3K4me3 and H3K27ac enrichment that were expressed in alveolar macrophages had significant (*P* < 0.05), greater than two fold overrepresentation for biological processes like viral protein processing, positive regulation of antigen receptor-mediated signaling pathway, type I interferon-mediated signaling, regulation of autophagy, and mitotic spindle assembly checkpoint, among others. Antigen processing and presentation via MHC class II was overrepresented at 1.76-fold (additional GO annotation in Supplementary Data 15). Motif analysis of promoter sequences identified many known bindings sites for transcription factor proteins ELF4, ETS, and interferon-regulatory factors (IRF1-3 and IRF8) within H3K4me3 regions. Additional *de novo* motifs had highest similarity to binding sites for transcription factor proteins SFPI1, MYB family, and CEBP family.

Further analysis of promoters revealed a small subset of 3,641 regions with signal enrichment for both H3K4me3 and the repressive mark H3K27me3. Annotation with the nearest gene revealed 45% of these regions were within 2 kb of a TSS. These 1,630 regions were considered bivalent promoters. Regions were associated with 1,166 protein-coding genes. Gene ontology analysis was largely enriched for genes involved in molecular functions for transcriptional regulation, transcription factor activity, DNA and RNA binding, and RNA polymerase II regulation (Figure 5B). Biological processes discovered in GO overrepresentation analysis involved cell and tissue differentiation, stimulus response, and cell movement, among others (Supplementary Data 15). Motif analysis revealed that *de novo* motifs were more significant (*P* = 10^–58^) than known motifs (*P* = 10^–30^) within bivalent promoter sequences. A *de novo* motif with similarity to the binding motif for yeast protein STB1 was found in 38% of regions. Known motif analysis revealed bivalent promoter sequences were enriched for “CCCGC” and “CGCGCG” sequences and the motif for the *Drosophila* GAGA factor protein.

### Enhancers Were Enriched for *de novo* Binding Motifs

Total enhancer regions with either H3K4me1, H3K27ac, or both were more numerous than H3K4me3 promoter regions. We found that genes have multiple enhancer regions. On average five significant regions enriched for enhancer signal were associated with each unique gene. In fact, we found that 69% of active enhancer regions marked with H3K27ac were found in clusters of two to seven (average of 3.2) significant regions around the same gene. Multiple regions meant that enhancers are further from the genes they control. H3K27ac regions were a mean of 1.9 kb further from their genes than H3K4me3 regions. Our data revealed that 56% of genes may be controlled by multiple active distal enhancers (e.g., H3K27ac regions further than 2 kb from genes) in macrophages.

**FIGURE 6:**
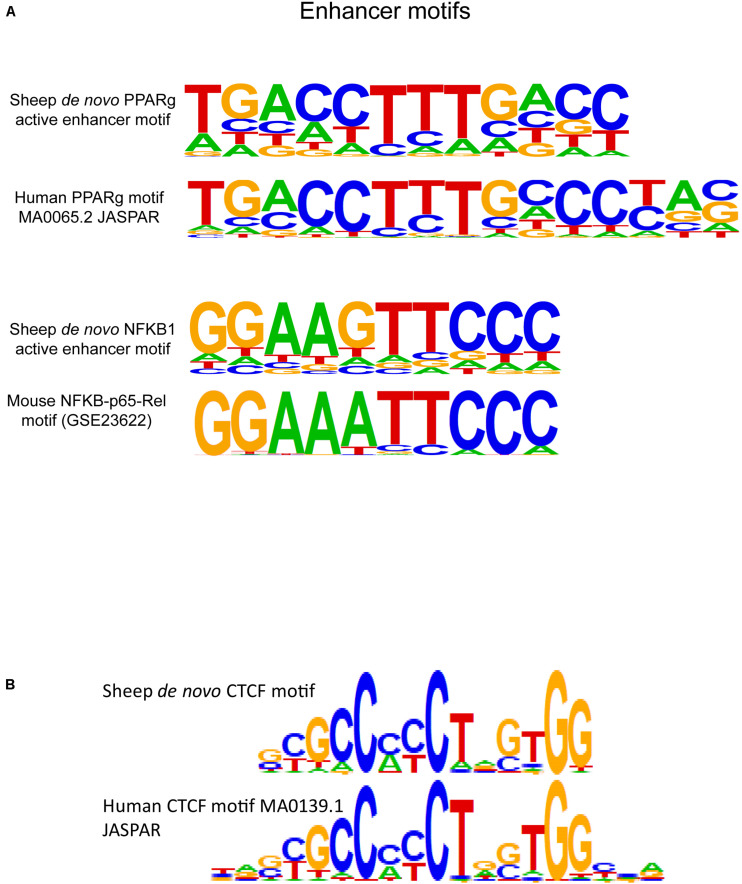
Regulatory element motif enrichment in sheep alveolar macrophages with comparison to known motifs from model species. **(A)** *De novo* motifs from sheep macrophages were discovered for the transcription factor protein PPARG in active enhancers (H3K27ac-enriched regions), this protein is a known tissue-specific regulatory protein in alveolar macrophages. The third motif is a *de novo* motif that had a 0.88 match score to NFKB1 protein binding motifs identified in mice. **(B)** HOMER scanning of insulator sequences identified by CTCF immunoprecipitation revealed enrichment in 50.5% of regulatory regions by the *de novo* motif in sheep displayed here. The motif is similar but not identical to the known CTCF motif from humans. Additional CTCF motifs discovered in sheep are shown in Supplementary Figure 12.

DNA sequences from active enhancer regions marked by H3K27ac were scanned for motifs with HOMER. Enhancer sequences contained similar central bases but often shorter consensus motifs with degenerate bases at the flanking sequences (Figure 6A) to known human macrophage-specific binding sites. Within the top three most significant *de novo* motifs within H3K27ac regions was a 15-bp sequence with 0.89 match score similarity to the known binding motif for the protein peroxisome proliferator activated receptor gamma (PPARG). PPARG is a transcription factor specific to macrophages within the lung microenvironment. The *de novo* PPARG motif was discovered in approximately 10% of active enhancers. Primed enhancers denoted by H3K4me1 were generally enriched for known lineage specific and pioneering factor motifs. Approximately half of H3K4me1 regions contained the known motif for the transcription factor protein PU.1. Primed enhancers in sheep additionally contained binding motifs for the transcription factor proteins CCAT enhancer binding protein beta (“C/EBP-beta,” CEBPB) and CEBPC, SpiB, and SpiC, and a *de novo* motif for NFKB1 reflecting presence of immunity related binding sites (Figure 6A).

### Silencers Were Associated With Lack of Gene Expression

Silencer regulatory elements denoted by H3K27me3 signal were generally not near genes on a genome-wide basis, since 69% of significant regions were in intergenic regions (see Supplementary Figure 11 for genomic localizations). Less than a third of H3K27me3 regions were within introns, and 1–2% of regions were within exons or near the 5′ end of genes. Under 7% of the genes nearest to H3K27me3 regions were expressed in alveolar macrophage RNA-seq data. Silencer regions were located an average of greater than 29 kb from the nearest gene. Motif analysis identified binding sites for two significant motifs (*P* < 10^–51^), to the proteins Zfp281 and NFKB1. Several additional C2H2 zinc finger protein binding sites were discovered with borderline significance (*P* = 10^–42^) for transcription factor proteins ZKSCAN1, ZNF467, and ZNF165. Silencer regions were found near homeobox transcription factor genes (*HOX* family) involved in embryologic development. The closest genes to H3K27me3 regions in the sheep genome included 46% of all homeobox genes know in humans.

### CTCF Insulators Motif Analysis and TAD Anchoring

Insulator element CTCF peaks were scanned with HOMER to determine the top *de novo* binding motifs in sheep and top known motifs (Figure 6B). CTCF and CTCF like motifs were the most significant motifs discovered amongst known motifs with (*P* = 10^–1482^) and amongst closest matches to *de novo* motifs (*P* = 10^–4338^) (Supplementary Figure 12). Significant motifs with match score greater than 0.75 were used to scan the DNA sequence of all CTCF regions and revealed 50.5% of consensus regions contained CTCF motifs. Next, we calculated the genomic distance between pairs of nearest CTCF enriched regions on each chromosome since these insulators form domains with paired anchors. We found that the genome could be divided into approximately 11,100 predicted TADs based on pair-wise counts per chromosome (see Supplementary Data 15). These regions were calculated to be an average of 258 kb in length. Based on gene content per chromosome insulators delimited an average of three genes per chromatin domain in sheep.

### HyperChIPable Regions Found in Sheep Macrophages

In our comparative analyses between ChIP-seq targets we discovered enriched signal in 1,520 regions of the genome that were significant compared with the control in all immunoprecipitated datasets. The regions did not have specificity for any antibody used for pulldowns. These “hyperChIPable regions” spanned a small fraction (0.43%) of the genome. However, they were broad at 8 kb, longer than regions found in any individual target. Although the majority of hyperChIPable regions were found within intergenic regions (59%), there was moderate but significant enrichment at promoters (*P* = 8.9 × 10^–8^) indicating a pattern to their location rather than pure noise. Like H3K4me3 regions, hyperChIPable regions were found within the first intron of genes or at the TSS (39%) (see Supplementary Figure 13).

## Discussion

### Overview

We identified repressive and active regulatory elements and validated regions by comparison to public RNA-seq data on alveolar macrophages from the Sheep Gene Expression Atlas (Clark et al., 2017). These data met the benchmarks for acceptable quality set forth by the ENCODE project consortium and the livestock FAANG consortium exceeding sequencing depth of 20 million usable fragments for narrow marks and 45 million usable fragments for broad marks with production of complex libraries (Dunham et al., 2012; Andersson et al., 2015). We identified the promoters and distal *cis*-acting regulatory elements for housekeeping genes, genes associated with macrophage differentiation, and tissue-specific alveolar macrophage genes. We found bivalent regulatory elements at the promoters of few genes and annotated GO processes that varied from the processes found in more typical promoters. We also identified significant regions bound by CTCF with N-ChIP that revealed insulators and allowed the first preliminary estimates of chromatin domains in sheep alveolar macrophages. Our collective data assigned a putative biological regulatory function in macrophages to nearly 12% of the sheep genome.

Alveolar macrophages served as a biologically interesting tissue given their myriad local functions and importance to zoonotic intracellular pathogens. We were able to identify GO overrepresentation in regulatory element associated genes involved in pathways reflective of tissue-resident macrophage main functions in homeostasis such as protein catabolism, autophagy, and nitrogen metabolism (Lavin et al., 2014). Both innate immune functions like interferon signaling, and adaptive immune functions like antigen processing and MHC class II presentation were identified as significant, reflecting macrophages unique role in both branches (Schmidt et al., 2016). We chose native ChIP-seq (N-ChIP) instead of formaldehyde cross-linked (X-ChIP) because it has been reported to preserve antibody epitopes leading to increased enrichment of signal and less back ground noise (O’Neill and Turner, 2003; Wagschal et al., 2007; Villar et al., 2015; David et al., 2017; Fang et al., 2019). Cells were frozen and stored short term prior to processing for N-ChIP since this method has been demonstrated to maintain sensitivity and reproducibility (Brind’Amour et al., 2015). While a possible limitation is that some protein-chromatin interactions may be lost in freezing. Optimization of shearing was effective with highly reproducible micrococcal nuclease digestion in our hands, that reliably yielded mononucleosomes. Native ChIP has compounded advantages in that no large protein-chromatin complexes are created that have been shown to inhibit shearing, and the endo- and exo-nuclease activity of micrococcal nuclease allows excellent resolution of the ChIP-seq target regions (Skene and Henikoff, 2015).

### Individual Chromatin Modifications in Sheep Macrophages Reflect Expectations

We found evidence in ChIP-seq enriched active regions (H3K4me3 and H3K27ac) of increased guanosine and cytosine content, as others have shown in various cell types that promotes open chromatin (Glass and Natoli, 2016). Heterochromatin and silencer sequences were shown to be depleted for GC content (Glass and Natoli, 2016) like our H3K27me3-enriched regions (see Table 2 for GC content summary). We found a clear bimodal distribution of active promoters and enhancers around the TSS (Figure 3) as has been reported by others for a variety of tissue types (Kingsley et al., 2020). At the TSS there was a slight depression in signal reflecting this nucleosome depletion that would allow for the positioning of the initiation complex and RNA polymerase along the chromatin.

Indicative of promoter regions, H3K4me3 enrichment should be detected at one-half to two-thirds of all genes in a cell including 60% of silenced genes (Barski et al., 2007). We were able to find reproducible peaks in both animal replicates that account for 50% of the annotated genes or pseudogenes in the sheep genome yielding putative active and primed (poised) promoters used in sheep alveolar macrophages. Promoters primed by H3K4me3 have also been demonstrated in macrophages notably at *TLR4* promoters and immediate early genes that help to induce rapid expression after exposure to foreign and injurious stimuli like LPS (Escoubet-Lozach et al., 2011). We produced an annotation resource for active regulatory elements at key immune genes including *TLR4, TLR8, TLR6*, MHC class II genes, *BHLHE40*, and *BHLHE41* that are highly expressed in alveolar macrophages (Supplementary Figure 10). The transcription factors BHLHE40 and BHLHE41 are master regulators that repress expression of lineage-inappropriate genes in alveolar macrophages and govern self-renewal. In fact, BHLHE40 inhibits H3K27ac in regulatory elements to control gene expression (Rauschmeier et al., 2019). *BHLHE41* was not annotated in the previous reference annotation for Oar_v3.1. Therefore its gene expression profile is absent from the original analysis in the Sheep Gene Expression Atlas, but it was associated with very high signal enrichment for ChIP-seq active marks so we would predict it is also highly expressed in sheep alveolar macrophages. Our analysis revealed regulatory elements for core tissue-specific genes like the transcription factor *PPARG* (Supplementary Figure 10) which regulates homeostasis and surfactant catabolism (Lavin et al., 2014) and also detected enrichment for its protein binding motif in active regulatory regions.

Nearly two-thirds of H3K4me3 regions in sheep macrophages were found further than 2 kb from annotated genes or within the first intron or first exon. This perhaps suggests that alternative start sites exist in macrophages or that gene and transcript annotation is incomplete for the sheep genome. Previous work showed that immunity related genes are enriched for tissue specific allelic expression (Salavati et al., 2019) so alveolar macrophages may express unique isoforms. The FR-AgENCODE project found similar results in immune cells of goats, where 37% of coding transcripts were determined to be alternative and multi-exonic (alternative splicing) compared to the reference annotations and many extensions of annotated genes were also discovered (Foissac et al., 2019). In fact, the ENCODE Project consortium originally detected a similar trend in humans and reported that the sole use of Refseq based annotations led to dramatically overestimated distance of regulatory elements from expected promoter locations at TSS (Birney et al., 2007). The annotation build for Oar_rambouillet_v1.0 included few immune tissues for gene prediction. Generation of experimental data to improve TSS annotation is one of the objectives defined by the Ovine FAANG Project and cap analysis gene expression (CAGE) data on 55 tissues and alveolar macrophages was recently published (Salavati et al., 2020). CAGE is an excellent method to confirm function and location of promoters and enhancers (Andersson et al., 2014) for validation of ChIP-seq (Wood et al., 2020).

Total enhancer regions were more numerous than active promoter regions as each gene can be controlled by multiple enhancers but generally a single promoter. Differentiation between active and primed distal regulatory elements was possible in our data as H3K27ac (Creyghton et al., 2010) had a clear association with sheep macrophage promoters and predicted gene expression from the Sheep Gene Expression Atlas. The mark H3K4me1 functions to prime enhancer regions disallowing recruitment of histone deacetylases and was less predictive of gene expression in sheep. Rather, we found these primed enhancer regions were highly enriched for canonical PU.1 binding motifs in sheep. So called pioneer factors, PU.1 can bind partially compact chromatin and help open chromatin for additional transcription factors (Bernstein et al., 2002). PU.1 is also a lineage-determining transcription factor highly active in macrophages (Glass and Natoli, 2016; Soares et al., 2017). We found PU.1 motifs in 50% of enhancers, Lavin et al. (2014) reported motifs in 30–40% of murine macrophage enhancers with X-ChIP. Sheep enhancers contained binding motifs for enhancer binding proteins CEBPB and CEBPD that are known to regulate genes involved in immunity including cytokines, chemokines, and proinflammatory factors (Wang et al., 2019). CEBP proteins also mediate acetylation of H3K27 through coactivators which prevent methylation at this residue, priming the region for further activation. Next, we identified insulators that can modulate enhancer function.

Our data was able to identify greater than 50,000 CTCF enriched regions in the genome of each individual animal and approximately 22,000 common to both animals. This matches the estimate of 40,000–50,000 CTCF occupied sites obtained in individual cell types from the wealth of ENCODE data (Ghirlando and Felsenfeld, 2016). This was an interesting experiment as relatively few studies use native chromatin for CTCF immunoprecipitation, and we may not have captured transient CTCF regions. In ChIA-PET studies, many insulators are transiently bound by CTCF with low correlation of occupancy and other regions form more permanent contacts, which may explain the lower percentage of overlapping sites we saw between the two sheep compared to other marks (Handoko et al., 2011; Guo et al., 2015). Native ChIP-seq is reported to be successful for CTCF since its binding affinity to chromatin is far greater than other transcription factors (Nakahashi et al., 2013). In fact, our dataset may be enriched for predominantly “non-exchangeable” CTCF sites that have the highest binding affinity and generally denote the largest structural chromosome loops. In the future, comparison with X-ChIP and Hi-C from sheep may be helpful to elucidate localized transient chromatin loops. We were able to estimate the average size of chromatin loops from our CTCF sites by empirically assuming pairs will form contact domains. This yielded a mean estimated domain size of 258 kb comparable to human contact domains determined from Hi-C data of median 185 kb (Rao et al., 2014). Literature surveys report 1,000–1,000,000 loops per genome (Fullwood et al., 2009; Jin et al., 2013; Sanborn et al., 2015). Our data yielded an estimated 11,000 pairs (22,000 regions) that could form loops in the sheep genome. Hi-C assays in goats yielded 8,990 TADs in goats with a similar size of 220 kb (Foissac et al., 2019).

We defined the first *de novo* CTCF motif in sheep macrophages. The central core of the 19-bp canonical motif is maintained between human and sheep, however, the flanking nucleotides on either end of the motif displayed heterogeneity compared to the core human motif in the JASPAR database (MA0139.1). The *de novo* motifs were found in approximately half of the sheep insulator regions, meaning half do not contain recognizable motifs or may have bindings sites adjacent to immunoprecipitated regions since CTCF binds chromatin in large protein complexes. However, from Rao et al. (2014), only 54% of CTCF-bound regions contain CTCF motifs, paralleling the 50% motif content we found in sheep. This can create difficulty in calling pairs of CTCF that form the anchors for TADs and localized sub-TADs.

### Combinatorial Patterns of Mark Overlap in Regulatory Elements

We found complex patterns of overlapping histone modifications across the regulatory element landscape. The “histone code” precisely titrates gene expression at multiple levels and is better assayed by analysis of multiple ChIP-seq targets together as we saw in sheep (Figure 2). This “cross-talk” reinforces the chromatin state by either supporting activation or attenuation of gene expression and may provide mechanisms for redundancy and epigenetic memory (Fischle et al., 2003; Wang et al., 2008). Epigenetic memory serves a key role in macrophages as it is the proposed mechanism behind trained immunity, that can reversibly recalibrate responses to pathogens, non-specifically. For all our ChIP-seq targets, regulatory elements contained at least some degree of overlap. Patterns of overlap in histone modifications compartmentalize the genome into euchromatin and heterochromatin, i.e., active versus repressed transcription. Subsequently, we saw relatively little overlap of H3K27me3 with the other marks tested as this is the only distinctly repressive mark we examined. In these sheep, the subset of active elements with both H3K4me3 and H3K27ac were better predictors of highly expressed genes that either mark alone, found at well-annotated genes as they were more frequently at the TSS. Conversely, we determined H3K27ac-exclusive regions were consistent with distal CREs (true enhancers) as reported in many species (Villar et al., 2015).

Some promoter regions in sheep macrophages were found to paradoxically have both H3K4me3 active marks and H3K27me3 repressive marks. These bivalent promoters signify unique genes that have highly variable and responsive gene expression (Vastenhouw and Schier, 2012). GO revealed different functions from those with active promoters with the caveat that both gene lists may contain noise from the RNA-seq data being from different animals than the ChIP-seq data. Alveolar macrophages are known to maintain tissue homeostasis when quiescent but once activated in response to invading pathogens or tissue injury can begin cytokinesis and phagocytosis (Lavin et al., 2014; Glass and Natoli, 2016; Schmidt et al., 2016). Bivalent promoters play essential roles in myeloid differentiation and when macrophage progenitors lose H3K27me3 repression at certain bivalent sites it can contribute to development of cancers like acute myeloid leukemia (Thalheim et al., 2017). Motif analysis revealed very few know transcription factor binding sites and several motif sequences of low complexity and high GC enrichment comparable to bivalent promoters of mammalian embryonic stem cells (Mantsoki et al., 2015).

In contrast, overlap of H3K27me3 and H3K27ac histone modifications are antagonistic to one another and not found in the same regions (Tie et al., 2009). Accordingly, we did not find enrichment of these two marks together in the same regions. Overall, silencer elements, H3K27me3, were found in broadly different locations than active elements captured by H3K27ac, H3K4me3 and H3K4me1. We were able to find enrichment of 6% of the sheep genome in alveolar macrophages with the silencer mark H3K27me3. This likely represents the bulk of this compartment in the sheep genome since H3K27me3 corresponds to regions of heterochromatin estimated to comprise 8% of the human genome as 92% is euchromatin (Consortium International Human Genome Sequencing, 2004; Rao et al., 2014). In our data, H3K4me1 and CTCF showed some overlap with one another and with H3K27me3 regions near boundary zones between heterochromatin and euchromatin. We found in sheep macrophages as Barski et al. (2007) found in human T-cells, that locations with CTCF enrichment also were enriched for multiple histone methylation marks found at domain boundaries.

Lastly, because we produced data for multiple marks, we were able to elucidate putative hyperChIPable regions in the sheep genome. These regions were found in all immunoprecipitated datasets and were not specific for any one target or antibody (Figure 3B). HyperChIPable regions were slightly more likely to be found at promoter regions and within the first intron of genes near TSSs (Supplementary Figure 13). This active promoter effect has been reported in the past for biologically hyperChIPable regions in human and mouse (Wreczycka et al., 2019). There may be a biological reason that these regions appeared in all immunoprecipitated fractions or are perhaps more efficiently sequenced. Enrichment of these non-specific sites near promoters may also be an artifact of the experimental protocol as micrococcal nuclease digestion is more efficient at euchromatin than heterochromatin, so a larger portion of fragment ends available for sequencing will naturally occur around open chromatin. HyperChIPable regions may also be caused by artifacts in the reference assembly. Regions containing repetitive elements are troublesome for genome assembly and may be collapsed, therefore natural copy number variation would create the appearance of falsely elevated signal in the region (Amemiya et al., 2019). We have provided these putative hyperChIPable regions for sheep in the public OSF repository (see section “Data Availability Statement”). As these regions were not known previously in sheep and not yet validated, we have not removed them from our ChIP-seq datasets. However, once validated in additional sheep tissues these regions can be included on a “block list” of sites to be removed from future experiments, like the ENCODE consortium created for model organisms, since they do not represent signal from the protein target of interest (Carroll et al., 2014).

### Regulatory Element Locations and Signal Enrichment Associate With Gene Expression

Generally, gene expression can be quantitatively predicted by the signal enrichment of histone modifications. However, specific gene expression is highly contingent on cell type and the usage of specific regulatory elements is cell type dependent, especially in immune genes (Lavin et al., 2014). Thus, it was critical for us to experimentally determine histone modifications in primary macrophages most representative of *in vivo* conditions rather than from cell culture conditions to identify the regulatory elements that are uniquely used by the immune system. We found modest, positive correlation that was highly statistically significant (*P* = 10^–130^), between signal enrichment of H3K4me3 in promoters and gene expression in alveolar macrophages determined in the Sheep Gene Expression Atlas (Clark et al., 2017). This correlation served as a “proof-of-concept” validation of our ChIP-seq regions. Importantly, we found that overlap of both H3K27ac and H3K4me3 had a stronger predictive value for gene expression than H3K4me3 alone as nearly 80% of genes with enrichment for both were expressed from RNA-seq. In sum these active regulatory elements were at the TSS of approximately 7,600 protein coding genes that were actively expressed in alveolar macrophages. Quantitative correlation between our ChIP-seq signal and RNA-seq expression was limited since the data was obtained from different animals, of different breeds, raised on different continents, and the RNA-seq data were obtain from two individual female animals in separate experiments. More complex regression analysis could improve correlation between ChIP-seq signal and RNA-seq data (Angelini and Costa, 2014), however, we opted for a simple analysis as proof-of-concept for this data resource. We would expect ChIP-seq signal to have improved quantitative correlation with RNA-seq data if generated from the same animals at the same time points. We envision the ChIP-seq data presented here being used as foundational annotation of CREs in quiescent macrophages from healthy sheep and these data will allow identification of target regions for further study. Future work may expand upon the multiple functions of macrophages by examining activated or infected macrophages and yield both epigenomic data and transcriptional data from the same macrophage populations. These types of studies have potential to capture epigenetic modifications caused by response to exogenous agents or orchestrated by infectious agents at regions identified in resting cells and at additional genomic regions (Hall et al., 2020; Herrera-Uribe et al., 2020).

We also captured transcriptional activators for a variety of types of RNA that could not be correlated to gene expression from mRNA-seq. For example, we identified the promoter for several members of the *let-7* microRNA precursor family. In human and murine macrophages, *let-7* has been shown to post-transcriptionally control cytokine production in innate immune responses by repressing production of interleukin-10 (IL-10), IL-6, and TLR4 until pathogens are detected (Schulte et al., 2011). Annotation of short RNA elements, which is largely missing from the sheep genome annotation, could be defined by combining RNA-seq methods with more stable DNA based methods like ChIP-seq to find short regions of active transcription. Our data indicated several regulatory elements that displayed the pattern of bona fide active promoters but are not near any currently annotated genes or regulatory RNA; we hypothesize these regions may control expression of either novel tissue-specific, short regulatory, or weakly expressed transcripts which are difficult to annotate. Deep sequencing RNA experiments in sheep and goats have indeed found lncRNA had shorter transcripts and weaker expression which explains difficulty in annotation of these types of functional elements (Clark et al., 2017).

### Conclusion

In summary, we generated ChIP-seq data for four core histone modifications and chromatin domain defining CTCF locations for the first time in sheep primary alveolar macrophages. We have shown that active enhancer and promoter signal enrichment was predictive of gene expression in sheep macrophages. We also provided annotations of novel hyperChIPable regions that may represent biological or non-specific experimental artifacts and potentially should be included on a “block list” to be removed from future ChIP-seq experiments in sheep. The data generated here are publicly available for researchers and will be valuable for comparative and ovine immunology studies as well as fine mapping to improve marker assisted selection for infectious disease resilience. ChIP-seq defined promoters may help to annotate TSSs of genes, especially those that are not well or widely expressed. We also put forth novel binding motifs found within regulatory elements in sheep macrophages. Understanding the epigenetic control and response mechanism of the immune system is very important not only for animal health and infectious agent eradication but also for numerous economically important production traits. The immune response in sheep has energy resource costs despite the health outcome, and this ultimately affects efficiency of meat and milk production for human consumption. Therefore, genetic, and epigenetic improvement of infectious disease resistance or tolerance is important to increasing production efficiency in sheep. Use of regulatory element annotation data to develop marker-assisted or genomic selection tools has advantages over traditional methods to control infectious diseases as it promotes selection of hardier animals prior to the introduction of pathogens and avoids antibiotic resistance altogether.

These data, as part of FAANG, can be readily incorporated into the reference genome annotation or viewed as custom tracks. Generation of these data on a macrophage immune cell type will allow future work on mutations and epigenetic variations that cause differences in sheep immune response, zoonoses transmission, and immunological effects on production efficiency.

## Data Availability Statement

The original contributions generated for this study are publicly available. The animal metadata are deposited to the EMBL-EBI BioSamples database (http://www.ebi.ac.uk/biosamples) under accession numbers SAMEA7423844 and SAMEA7423845 and samples under SAMEA7423964 and SAMEA7423965. Raw DNA sequencing data in .fastq.gz format is available on the FAANG data portal and within European Nucleotide Archive (ENA) project accession PRJEB40528 (https://www.ebi.ac.uk/ena/browser/view/PRJEB40528). Analysis BED files of the peak calls in each animal, the consensus regions for each mark, bivalent promoters regions and hyperChIPable regions are available on the Open Science Framework website upon request and will be made public, https://osf.io/8rq4c/?view_only=1937b862ea874a14b85fff2043a9b522. Publicly available datasets from the Sheep Gene Expression Atlas (Roslin Sheep Atlas) were analyzed in this study. This data can be found here: University of Edinburgh DataShare portal at http://dx.doi.org/10.7488/ds/2112 (Clark et al., 2017). The sheep reference genome Oar_rambouillet_v1.0 is available at NCBI under accession GCA_002742125.1 (https://www.ncbi.nlm.nih.gov/assembly/GCF_002742125.1/) (Worley, 2017).

## Ethics Statement

The animal study was reviewed and approved by Institutional Animal Care and Use Committee at Washington State University.

## Author Contributions

Experimental protocols were designed by AM, MM, MH, BM, and SW. Cell collection was completed by AM, MM, and MH. Experiments, data generation, and initial data analysis were completed by AM. The bioinformatics pipeline was designed by AM and DH. Final analysis was completed by AM, MM, MH, DH, BM, and SW. AM, MM, BM, and SW acquired funding. AM wrote the first draft of the manuscript and created visualizations. All authors contributed to editing the manuscript and visualizations. All authors read and approved the final manuscript.

## Conflict of Interest

The authors declare that the research was conducted in the absence of any commercial or financial relationships that could be construed as a potential conflict of interest.

## Footnotes

1 https://data.faang.org/api/fire_api/assays/WSU_SOP_Native_ChIP-seq_Protocol_2019.pdf

2 http://broadinstitute.github.io/picard/

3 http://www.ebi.ac.uk/ena/data/view/PRJEB19199

4 http://www.ebi.ac.uk/ena/data/view/PRJEB6169

## Abbreviations

ChIP-seq: chromatin immunoprecipitation with sequencing
CREs: *cis*-regulatory elements
DPBS: Dulbecco’s phosphate buffered saline
ENCODE: Encyclopedia of DNA elements Project Consortium
FAANG: Functional Annotation of Animal Genomes Consortium
FDR: false discovery rate
FRiP: fraction of reads in peaks
GO: gene ontology
H3K4me1: histone three lysine four monomethylation
H3K4me3: histone three lysine four trimethylation
H3K27ac: histone three lysine 27 acetylation
H3K27me3: histone three lysine 27 trimethylation
kb: kilobases
N-ChIP: native chromatin immunoprecipitation with sequencing
NSC: normalized strand correlation
RSC: relative strand correlation
TAD: topologically associating domain
TPM: transcripts per million
TSS: transcription start site
X-ChIP: cross-linked chromatin immunoprecipitation with sequencing.
