# Supplementary material for: Genome-Wide Histone Modifications and CTCF Enrichment Predict Gene Expression in Sheep Macrophages
Source: Front Genet. 2021 Jan 7;11:612031. doi: 10.3389/fgene.2020.612031 (PMC7817998; doi:10.3389/fgene.2020.612031)

Supplementary Data

S1-S14

S1 Supplementary Methods, text.	Page 3
S2 Table. ChIP-seq antibody provider and lots.	Page 6
S3 Table. Bioinformatic analysis parameters.	Page 7
S4 Supplementary Results, text.	Page 8
S5 Figure. Pearson and Spearman heatmaps.	Page 9
S6 Figure. Scaled Venn diagrams of replicate peaks.	Page 10
S7 Figure. Fingerprint plots for ChIP-seq enrichment.	Page 11
S8 Figure. Total peaks for each animal replicate.	Page 12
S9 Figure. Pie charts genome comparison, H3K4me3.	Page 13
S10 Figure. ChIP-seq signal at Immunity-related genes.	Page 14
S11 Figure. Genomic annotation category, H3K27me3.	Page 16
S12 Figure. CTCF motifs from HOMER found in sheep.	Page 17
S13 Figure. HyperChIPable regions by genomic category.	Page 18
S14 Figure. ChIP-seq signal at high resolution.	Page 19

S1. Supplementary Methods

Cell collection

Sheep were chosen for mixed-breed heritage from the most common breeds found in production flocks in the Western United States. Both have known pedigrees and are half siblings by the same Targhee x Polypay sire. Dams were crossbreeds (Columbia, Polypay, Rambouillet, Suffolk, Targhee). Animals were fed normally (free choice grass and hay) prior to euthanasia and were removed from the outdoor pasture enclosure and transported to the necropsy room approximately an hour prior to euthanasia and group housed until euthanasia. In detail, trans-tracheal bronchoalveolar lavage fluid was collected within 20 minutes of euthanasia by three serial lavages with one liter sterile Dulbecco's phosphate buffered saline (DPBS, Mg^{2+} Ca^{2+} free) and gentle massage of the pulmonary parenchyma prior to decanting. Cells were isolated from collected lavage fluid by centrifugation (400 x *g* for 10 minutes) and washed twice with DPBS at room temperature. Erythrocytes within the pellet were lysed by suspension for 30 seconds in sterile water followed by quenching with 10X concentrated DPBS to restore isotonicity.

Frozen cells were thawed on ice with the addition of 1 mL of ice cold DPBS. Glycerin and cryopreservative were removed from the cells by two washes with 1 mL ice cold DPBS and cells collected by brief centrifugation at 4 C, 350 x *g*. Cells were then resuspended in hypotonic buffer for isolation of native chromatin. Chromatin digestion was optimized with a time trial experiment on sheep alveolar macrophages from additional animals prior to digestion on the two ewes presented here.

Additional details are provided on the FAANG data portal protocols for this experiment available here https://data.faang.org/api/fire_api/assays/WSU_SOP_Native_ChIP-seq_Protocol_2019.pdf

Bioinformatics

Detailed description including example scripts used to process data are provided on the FAANG data portal here https://data.faang.org/api/fire_api/assays/WSU_SOP_ChIP-seq_Bioinformatic_Analysis_Protocol_2020.pdf

BWA aln and samse commands were used sequentially for mapping. Data mapped to the Rambouillet genome (Oar_Rambouillet_v1.0) was used for downstream analysis; reads were also separately mapped to the previous genome versions Oav_v3.1 and Oar_v4.0 (Oar_v3.1 GCA_000298735.1 (ISGC, 2012), Oar_v4.0 GCA_000298735.2 (ISGC, 2015), see Supplementary Table S2 excel file). Mapping rates between datasets for the various genome reference versions were compared using Student's t-test (Supplementary Table S2 excel file). Reads were filtered for quality and unique mapping utilizing a mapQ cutoff score of 5 with samtools v1.9 (Li, H, 2009) for downstream analysis. Mapped reads were also processed with filtering by various mapQ scores from 1 to 30. The Golden Path length estimate for effective genome size takes into account the short read length of 50 bp and MAPQ filter applied. Peaks for all datasets were called at multiple false discovery rates of less than 1%, 5%, and 10%. MACS2 broad mode uses a default setting of 0.1 FDR to call broad peaks and the specified FDR to call

narrow peaks. Quality metrics as recommended by ENCODE standards (Landt et al., 2012) including fraction of reads in peaks (FRiP), non-redundant fraction, NSC, and RSC, are provided in the Supplementary excel file .

To find overlapping peaks, the non-directional union of all peaks in the three datasets was found with bedops, and then filtered to keep only those peaks that were discovered, defined by any overlap, in all three datasets; thus removing peaks that were only discovered in one animal or not sufficiently enriched in either individual animal (*i.e.* peaks only called in the pooled dataset), see Supplementary Table S3 for tool options.

Consensus peak overlaps between marks, displayed in Figure 2, were identified by using a combination of intersecting peak discovery in relation to the mark of interest (bedfile -a) with another dataset (bedfile -b) in bedtools, then selecting for those peaks retained in this simple two way overlap that are or are not also marked by the remaining three datasets (*i.e.* negatively selecting by additional overlap with 1, 2, or 3 remaining datasets) with bedops. Read depth and coverage within peaks was obtained by bedtools coverage with reference to the consensus peak bed file overlaying the quality filtered BAM (with duplicates removed) from the pooled reads.

deepTools bamCoverage was used to convert aligned and filtered BAM files for each dataset into .bigwig format with normalization of read depth using RPGC that scales to 1X read depth based on effective genome size. bigwigCompare was used to subtract the input signal from the immunoprecipitated datasets in order to view tracks as enriched signal above the control. Reads were extended to the approximate fragment length of 150 base pairs and signal bins were set to 10 base pairs. Tracks were viewed in IGV or IGB (Freese et al., 2016; Thorvaldsdóttir et al., 2013).

CTCF motif analysis. Using HOMER, de novo and known motifs were discovered within 100 bp of the consensus peak summit. The top matches amongst known motifs included CTCF(Zf)/CD4+-CTCF-ChIP-Seq(Barski_et_al.) and BORIS(Zf)/K562-CTCFL-ChIP-Seq(GSE32465), the full length motif for these were found in 16.9% (*P*= 1e-1934) and 16.8% (*P*= 1e-1482) of the sheep target sequences. The top de novo motif, most similar to BORIS CTCF-like zinc finger protein motif, CTCF canonical human motif from JASPAR, the CTCF motif discovered in CD4+ cells, and the CTCFL canonical motif from JASPAR, and its reverse opposite motif were used for motif scanning of the sheep CTCF reproducible consensus peaks. Motif scanning also included matrices from the top 11 degenerate or partial motifs with greater than 0.75 similarity to the top de novo motif and that had p values below 1e-728. These compiled motif matrices in addition to the human canonical CTCF motif from JASPAR MA0139.1 for a total of 16 motifs that were variable lengths of the same core motif, were fed back into HOMER to call the total CTCF peaks that contain the relevant CTCF motifs with high match scores for known CTCF motifs.

Processed data from Clark, et al. (2017) Supplementary "S1 dataset: Gene expression level atlas as transcripts per million (unaveraged)" was downloaded and filtered. Firstly, mitochondrial genes were removed as they will not be represented in ChIP-seq. RNA-seq expressed genes with an average TPM >1 within alveolar macrophages or a TPM above one in any individual animal from the above animal replicates were used which included 12420 protein-coding genes and 75

pseudogenes denoted as the superset of discoverable expressed genes in sheep alveolar macrophages. Further refinement of the two female replicates yielded 11641 genes or pseudogenes above an average TPM of 1 which would be expected to be discoverable in any single female sheep alveolar macrophage expression analysis which included two animal replicates, matched to our ChIP-seq experimental design. A list of the ranked gene names used for correlation analysis to ChIP-seq is given in Supplementary Data Excel file S15.

References

Clark, E. L., Bush, S. J., McCulloch, M. E. B., Farquhar, I. L., Young, R., Lefevre, L., Pridans, C., Tsang, H. G., Wu, C., Afrasiabi, C., Watson, M., Whitelaw, C. B., Freeman, T. C., Summers, K. M., Archibald, A. L., & Hume, D. A. (2017). A high resolution atlas of gene expression in the domestic sheep (Ovis aries). *PLOS Genetics*, *13*(9), e1006997. https://doi.org/10.1371/journal.pgen.1006997

Consortium, I. S. G. (2012). *Oar_v3.1*. GCA_000298735.1. https://www.ncbi.nlm.nih.gov/assembly/GCF_000298735.1/

Consortium, I. S. G. (2015). *Oar_v4.0*. GCA_000298735.2. https://www.ncbi.nlm.nih.gov/assembly/GCF_000298735.2/

Freese, N. H., Norris, D. C., & Loraine, A. E. (2016). Integrated genome browser: visual analytics platform for genomics. *Bioinformatics (Oxford, England)*, *32*(14), 2089–2095. https://doi.org/10.1093/bioinformatics/btw069

Landt, S. G., Marinov, G. K., Kundaje, A., Kheradpour, P., Pauli, F., Batzoglou, S., Bernstein, B. E., Bickel, P., Brown, J. B., Cayting, P., Chen, Y., DeSalvo, G., Epstein, C., Fisher-Aylor, K. I., Euskirchen, G., Gerstein, M., Gertz, J., Hartemink, A. J., Hoffman, M. M., … Snyder, M. (2012). ChIP-seq guidelines and practices of the ENCODE and modENCODE consortia. *Genome Research* , *22*(9), 1813–1831. https://doi.org/10.1101/gr.136184.111

Thorvaldsdóttir, H., Robinson, J. T., & Mesirov, J. P. (2013). Integrative Genomics Viewer (IGV): high-performance genomics data visualization and exploration. *Briefings in Bioinformatics*, *14*(2), 178–192. https://doi.org/10.1093/bib/bbs017

S2. Supplementary Table of Antibodies used for each ChIP-seq target. The company, catalog number, and lot number are provided for each ChIP-seq antibody. These antibodies have been validated in other species to ENCODE standards with two independent methods. The lot used for sheep experiments was validated with low level sequencing to ensure antibody-specific enrichment.

Antibody target	Provider and catalog number	Lot #	Description	References
H3K4me3	Abcam ab8580	GR3221448-1	Rabbit polyclonal	(2) (3)
H3K27ac	Abcam ab4729	GR3211959-1	Rabbit polyclonal	(1) (3)
H3K27me3	Millipore 07-449	3018864	Rabbit polyclonal	(1) (4)
H3K4me1	Millipore 07-436	2975223	Rabbit polyclonal	(1) (5)
CTCF	Cell Signaling 3418S (D31H2)	Lot 3	Rabbit monoclonal	(6)

1. Egelhofer TA, Minoda A, Klugman S, Lee K, Kolasinska-Zwierz P, Alekseyenko AA, Cheung MS, Day DS, Gadel S, Gorchakov AA, Gu T, Kharchenko PV, Kuan S, Latorre I, Linder-Basso D, Luu Y, Ngo Q, Perry M, Rechtsteiner A, Riddle NC, Schwartz YB, Shanower GA, Vielle A, Ahringer J, Elgin SC, Kuroda MI, Pirrotta V, Ren B, Strome S, Park PJ, Karpen GH, Hawkins RD, Lieb JD. An assessment of histone-modification antibody quality. *Nat Struct Mol Biol*. 2011 Jan;18(1):91-3. doi: 10.1038/nsmb.1972. Epub 2010 Dec 5. PMID: 21131980; PMCID: PMC3017233.
2. Liu Y, Han D, Han Y, Yan Z, Xie B, Li J, Qiao N, Hu H, Khaitovich P, Gao Y, Han JD. Ab initio identification of transcription start sites in the Rhesus macaque genome by histone modification and RNA-Seq. Nucleic Acids Res. 2011 Mar;39(4):1408-18. doi: 10.1093/nar/gkq956. Epub 2010 Oct 14. PMID: 20952408; PMCID: PMC3045608.
3. Ram O, Goren A, Amit I, Shoresh N, Yosef N, Ernst J, Kellis M, Gymrek M, Issner R, Coyne M, Durham T, Zhang X, Donaghey J, Epstein CB, Regev A, Bernstein BE. Combinatorial patterning of chromatin regulators uncovered by genome-wide location analysis in human cells. Cell. 2011 Dec 23;147(7):1628-39. doi: 10.1016/j.cell.2011.09.057. PMID: 22196736; PMCID: PMC3312319.
4. Wang H, Zou J, Zhao B, Johannsen E, Ashworth T, Wong H, Pear WS, Jonathan Schug, Stephen C. Blacklow, Kelly L. Arnett, Bradley E. Bernstein, Elliott Kieff, Jon C. Aster. Genome-wide analysis reveals conserved and divergent features of Notch1/RBPJ binding in human and murine T-lymphoblastic leukemia cells. *PNAS.* Sep 2011, 108 (36) 14908-14913; doi: 10.1073/pnas.1109023108
5. Zhou X, Cain CE, Myrthil M, Lewellen N, Michelini K, Davenport ER, Stephens M, Pritchard JK, Gilad Y. Epigenetic modifications are associated with inter-species gene expression variation in primates. Genome Biol. 2014;15(12):547. doi: 10.1186/s13059-014-0547-3. PMID: 25468404; PMCID: PMC4290387.
6. Guertin MJ, Cullen AE, Markowetz F, Holding AN. Parallel factor ChIP provides essential internal control for quantitative differential ChIP-seq. Nucleic Acids Res. 2018;46(12):e75. doi:10.1093/nar/gky252

Supplementary Table S3. Bioinformatics tools and parameters.

Tool	Tool version	Parameters
bcl2fastq2	v2.17.1.14	default parameters
fastQC	v0.11.3	default parameters
bwa	v0.7.17	aln, -q 15, and -t 24<br>samse, default parameters
samtools	v1.9	view, -b -q 15<br>index, default parameters
Picard	v2.9.2	SORT_ORDER=coordinate,<br>CREATE_INDEX=true,<br>VALIDATION_STRINGENCY=LENIENT
MACS2	v2.1.1.20160309	callpeaks, -g 2.62e9, -B -q 0.05<br>for broad peaks: -broad
HOMER	v4.10.4	annotatePeaks.pl, -annStat<br>findMotifs.pl,<br>for CTCF: -S 10<br>for others: -size given -S 15
deepTools	v3.3.0	multiBamSummary, bins<br><br>plotCorrelation, --corMethod spearman<br>--corMethod pearson<br><br>plotFingerprint, --ignoreDuplicates<br>--extendReads 160 --centerReads<br><br>bamCoverage, --binSize 10<br>--normalizeUsing RPGC<br>--effectiveGenomeSize 2620000000<br>--extendReads 150<br><br>bigwigCompare, --binSize 10 --bigwig1 ChIPFile.bw --bigwig2 ControlFile.bw -of bigwig --operation subtract
bedtools	v2.26.0	intersectBed, -wa -u -sorted

S4. Supplementary results

Mean mapping rates for raw reads was improved between updated versions of the sheep reference genome from 97.06% for OAR v3.1, 97.19% for OAR v4.0, and 98.58% for the current reference assembly available on NCBI, Rambouillet _v1 (see Supplementary Tabular File S15) [Oar_v3.1 GCA_000298735.1, Oar_v4.0 GCA_000298735.2, Oar_rambouillet_v1.0 GCA_002742125.1]. Thus, the current Rambouillet reference assembly was chosen for downstream analysis.

Interestingly, the genomic regions identified as active promoters had lower mapping rates to the previous two versions of the sheep reference genome than all other ChIP marks. This difference in promoter mapping rate was not apparent in the Rambouillet reference assembly that includes long read sequencing.

Mapping to different reference genomes had a more significant effect on number of genome-wide peaks. While individual animal peak calls can change by as much as 27% based on FDR reproducible consensus peaks are not perturbed by changing the FDR cut off between 5 and 10%. Therefore, the peaks we are calling are robust and not greatly dependent on FDR.

Supplementary Figure S5. Pearson and Spearman correlation heatmaps between all mapped read signal pileup. Data sorts by mark rather than by animal. Numerical correlation matrices are given in Supplementary Tabular File 1.

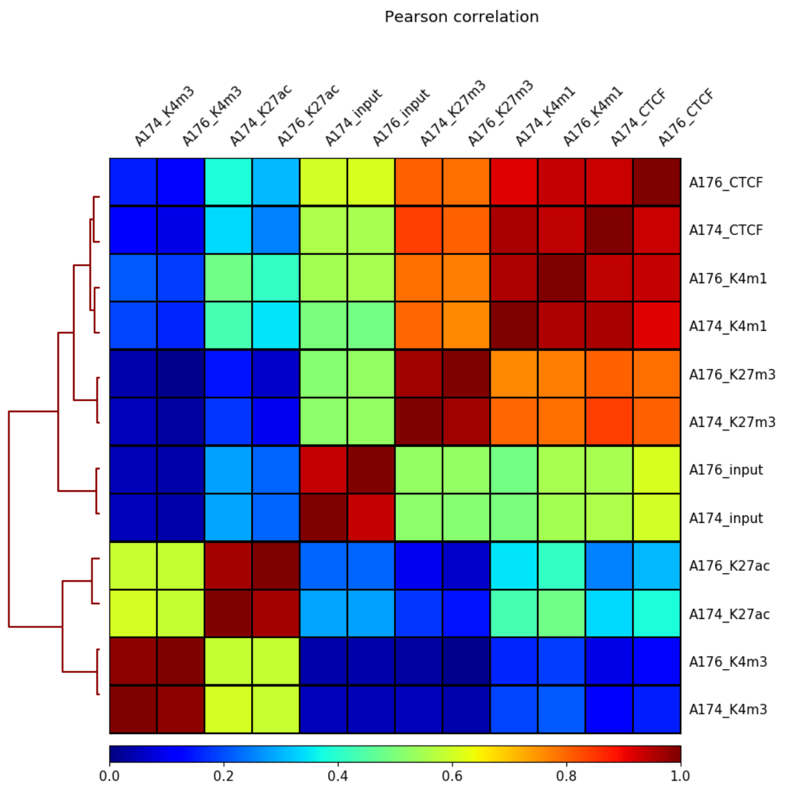

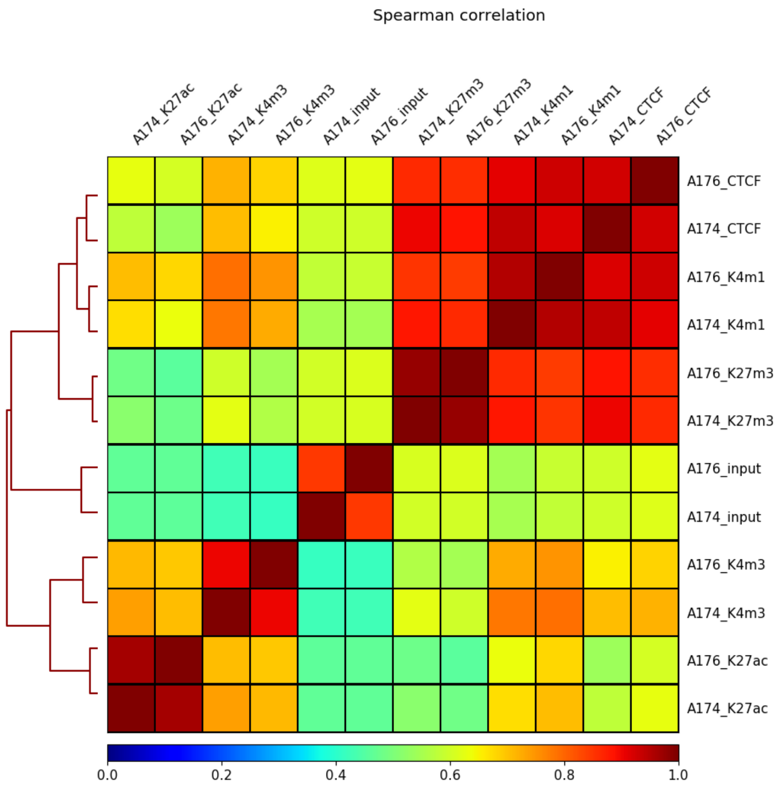

Supplementary Figure S6. Shared peaks identified between animal replicates. Scaled Venn diagrams of reproducible peaks between the two animal replicates. Displays the average overlap of peaks between the two animal replicates for each mark.

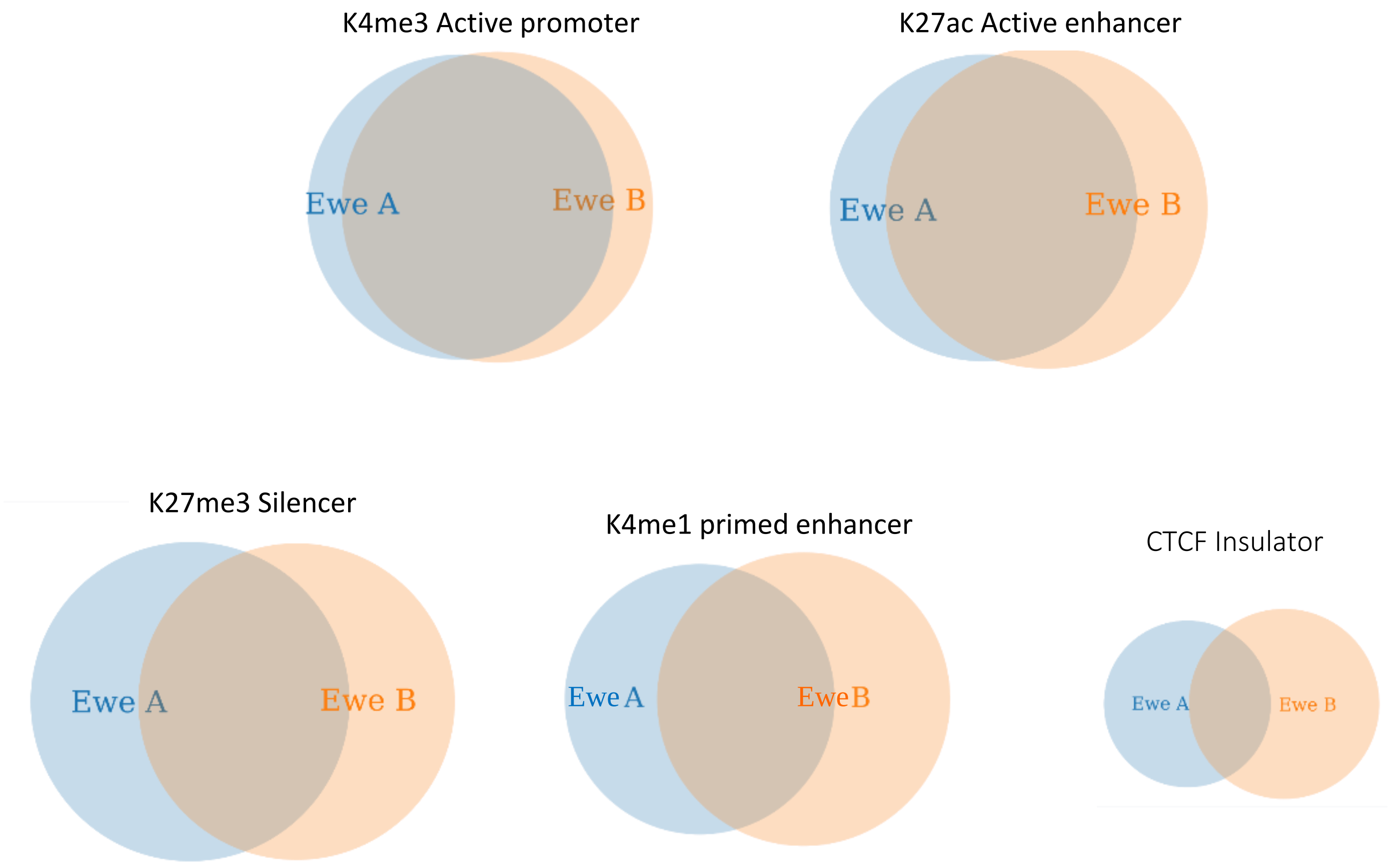

Supplementary Figure S7. Cumulative enrichment fingerprint plots. Fingerprint plots for ChIP-seq enrichment. **(A)** All marks **(B)** Narrow marks H3K4me3, H3K27ac, and CTCF **(C)** Broad marks H3K27me3 and H3K4me1.

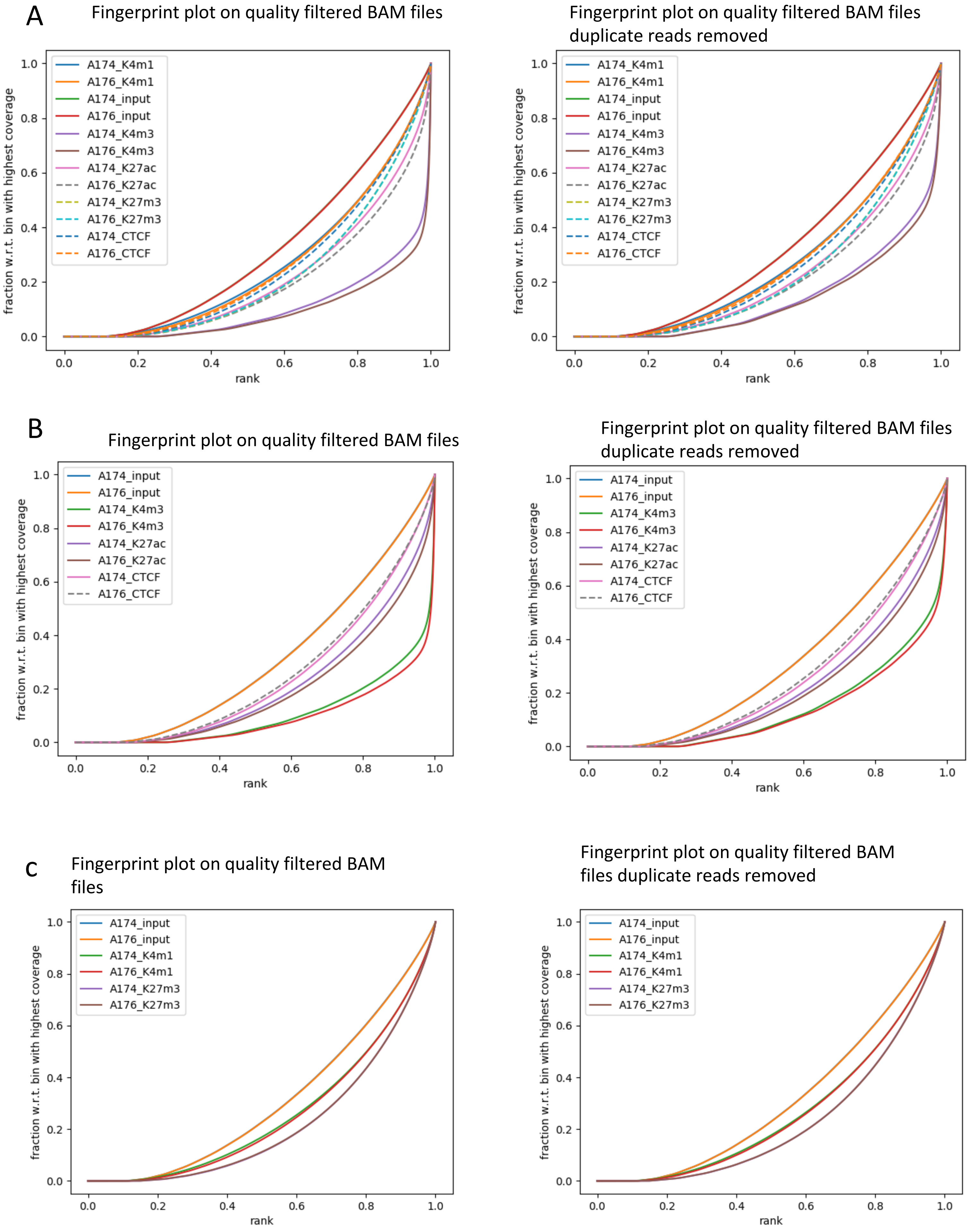

Supplementary Figure S8. Total number of regulatory element regions (peaks) for each animal and in consensus tracks**.** Bar charts representing the number of peaks found in each individual animal and displaying the total consensus peaks that were found in both animals and also called in pooled reads from both animals, false discovery rate cut off of 5%.

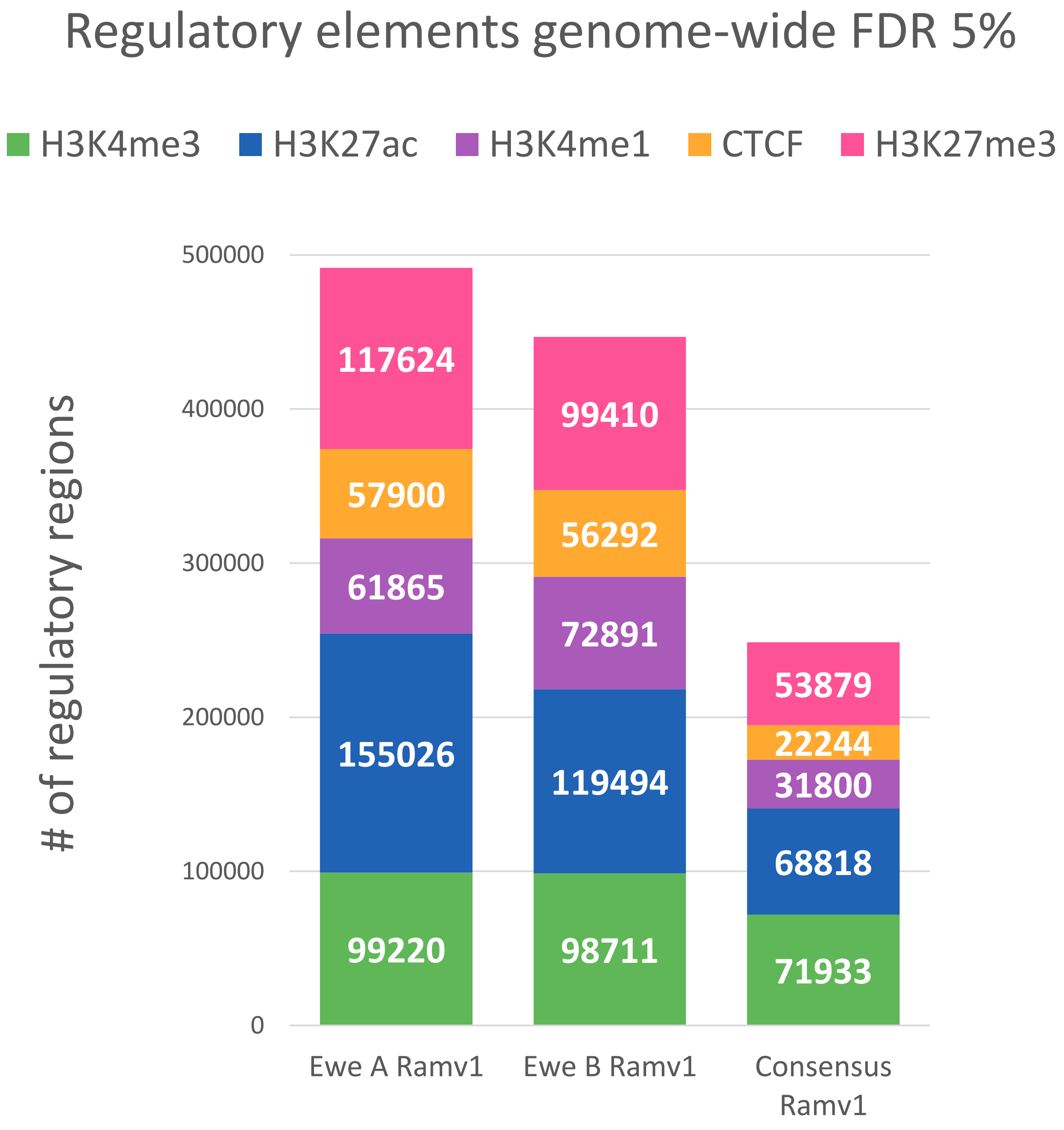

A

B

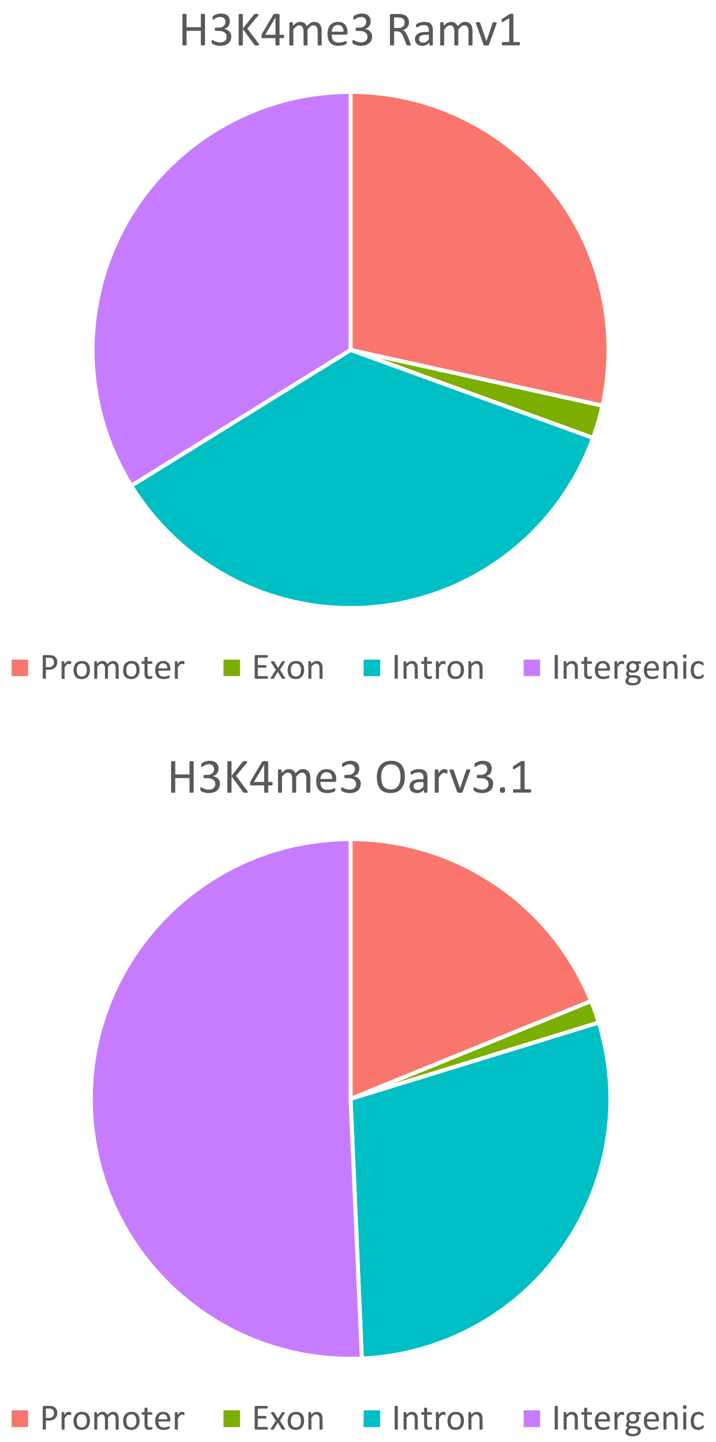

Supplementary Figure S9. Comparison of binned categories where H3K4me3 enriched signal occurs between reference genomes. Promoter is defined as +/- 2 Kb of the transcription start site. **(A)** Oar_rambouillet_v1.0, and **(B)** Oar_v3.1.

Supplementary Figure S10

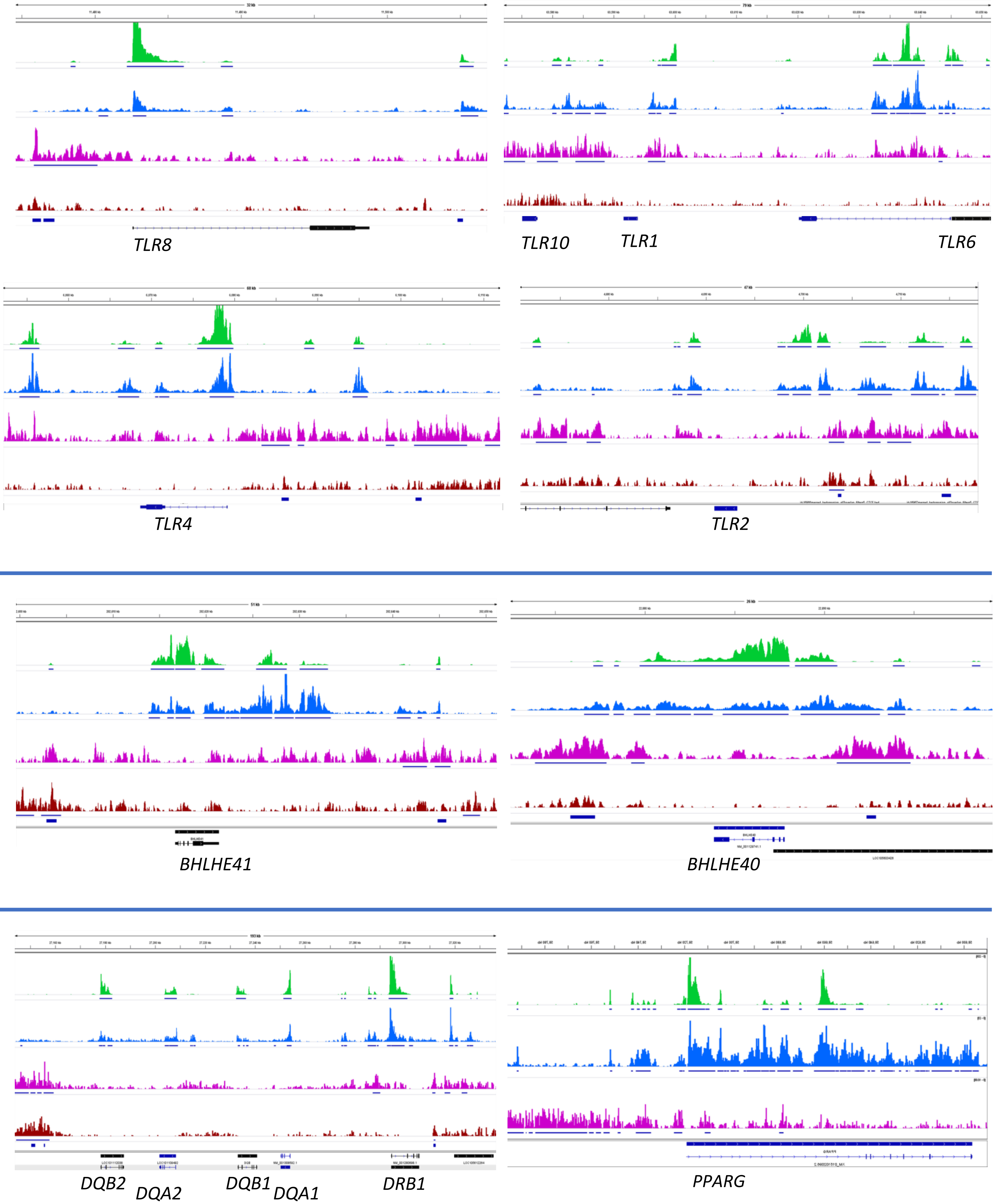

Supplementary Figure S10. Extended tracks of ChIP-seq peaks around immunity-related genes. Includes blue bars beneath the signal tracks representative of regions with significant signal called by MACS2 and present in consensus peaks. The Y-axis signal is displayed in RPGC with input signal subtracted and the X-axis is distance/location along the linear chromosome. On the top are toll-like receptor genes expressed in alveolar macrophages. In the middle are the key transcription factor genes for *BHLHE40/41* which control alveolar macrophage differentiation and cytokine response. On the bottom are the MHC class II genes and *PPARG* a key tissue-specific gene that controls catabolism of surfactant in the lung microenvironment.

Supplementary Figure S11. Genomic annotations of silencer regions by category. The pie chart displays the default annotations from HOMER. Promoter is defined as +/- 500 bp of an annotated transcription start site. Statistical enrichment at each category for H3K27me3 as calculated by HOMER.

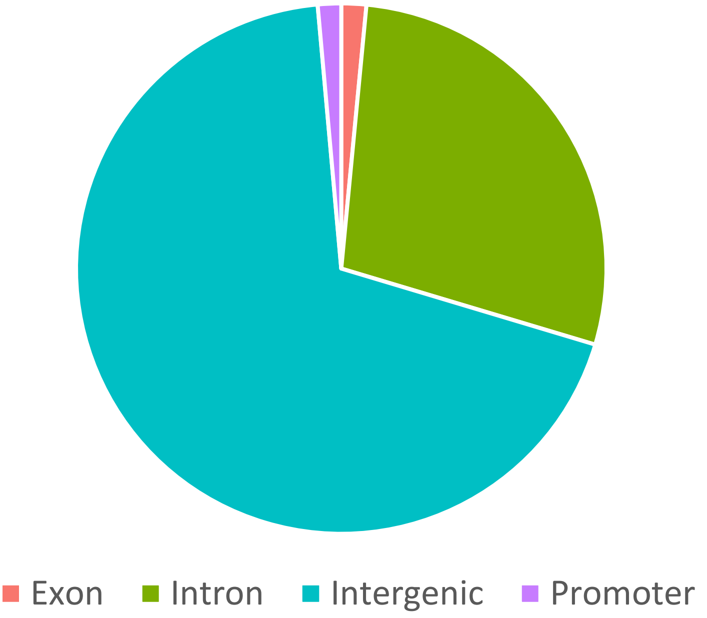

Annotation	Number of peaks	Total size (bp)	Log2 Ratio (obs/exp)	LogP enrichment (+values depleted)
TTS	633	3.10E+07	0.167	-6.145
Exon	768	6.08E+07	-0.526	62.169
Intron	14204	1.01E+09	-0.371	769.665
Intergenic	34840	1.68E+09	0.192	-794.562
Promoter	719	3.51E+07	0.171	-7.048

Supplementary Figure S12. CTCF motifs from HOMER. (A) Additional known motif matches to the top motif identified in sheep CTCF peaks. **(B)** *de novo* motifs found in sheep CTCF peaks and the most similar match to known binding site motifs.

A

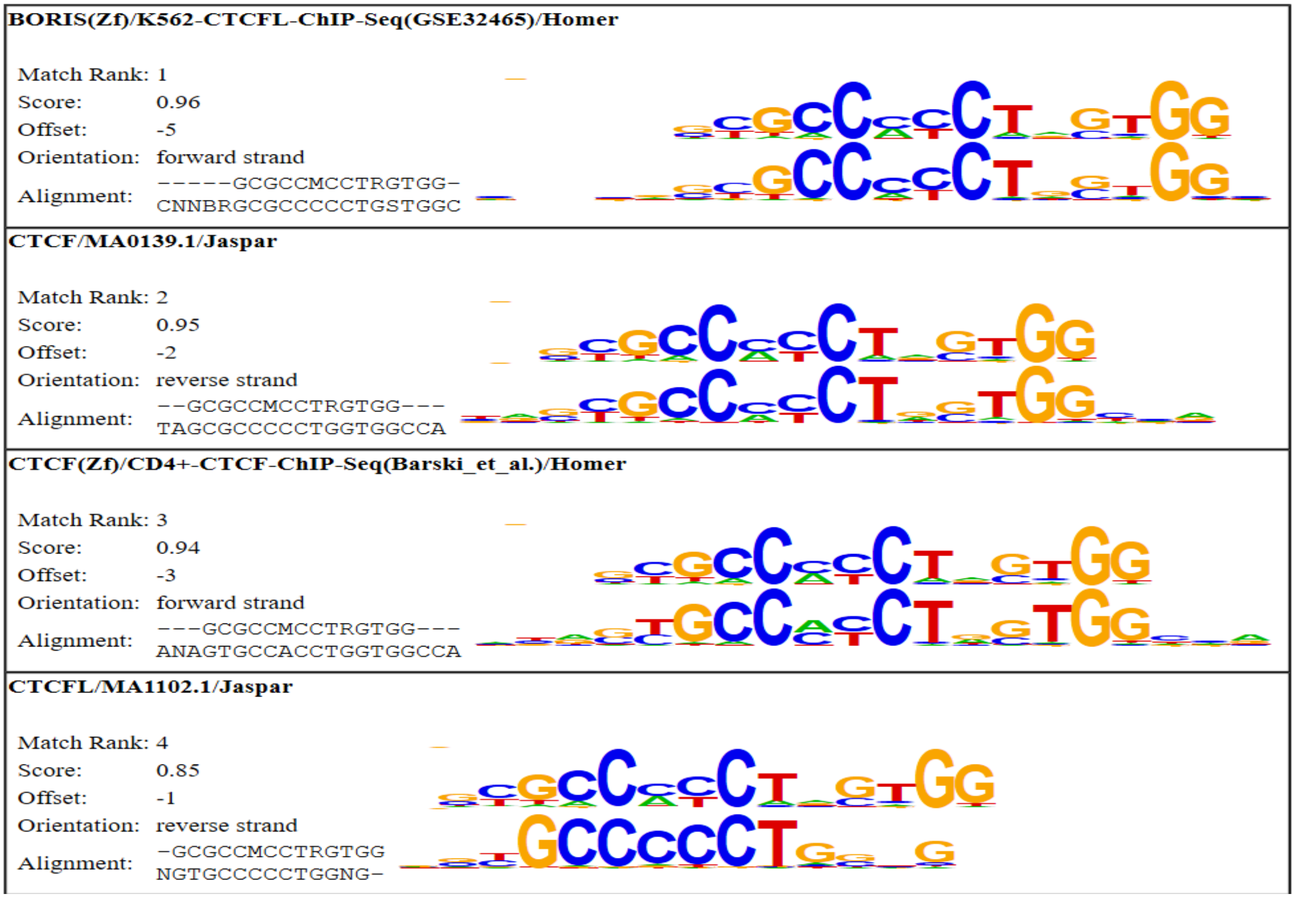

B

Homer *de novo* Motif Results

Rank	Motif	P-value	log P-pvalue	% of Targets	% of Background	STD(Bg STD)	Best Match/Details
1	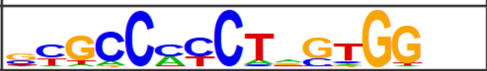	1e-4338	-9.989e+03	7.43%	0.01%	14.2bp (27.2bp)	BORIS(Zf)/K562-CTCFL-ChIP-Seq(GSE32465)/Homer(0.964) More Information | Similar Motifs Found
2	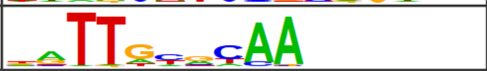	1e-144	-3.336e+02	3.52%	1.22%	23.7bp (29.6bp)	CEBP(bZIP)/ThioMac-CEBPb-ChIP-Seq(GSE21512)/Homer(0.945) More Information | Similar Motifs Found
3	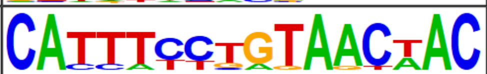	1e-109	-2.519e+02	0.32%	0.01%	18.3bp (0.0bp)	EWS:ERG-fusion(ETS)/CADO_ES1-EWS:ERG-ChIP-Seq(SRA014231)/Homer(0.636) More Information | Similar Motifs Found
4	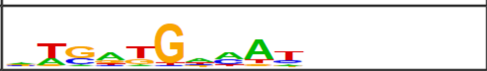	1e-87	-2.023e+02	4.01%	1.92%	26.7bp (27.2bp)	Chop(bZIP)/MEF-Chop-ChIP-Seq(GSE35681)/Homer(0.852) More Information | Similar Motifs Found
5	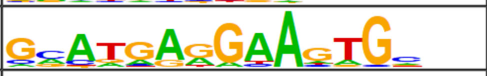	1e-76	-1.769e+02	0.29%	0.01%	21.0bp (14.5bp)	PU.1(ETS)/ThioMac-PU.1-ChIP-Seq(GSE21512)/Homer(0.808) More Information | Similar Motifs Found
6	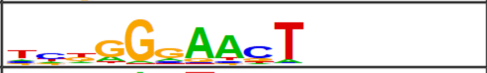	1e-66	-1.524e+02	10.78%	7.57%	26.7bp (26.6bp)	RBPJ/MA1116.1/Jaspar(0.734) More Information | Similar Motifs Found
7	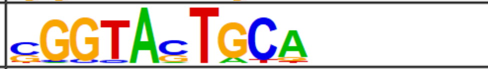	1e-63	-1.455e+02	0.21%	0.00%	22.6bp (0.0bp)	Ddit3::Cebpa/MA0019.1/Jaspar(0.740) More Information | Similar Motifs Found
8	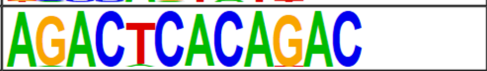	1e-61	-1.411e+02	0.24%	0.01%	24.6bp (15.4bp)	ARG80(MacIsaac)/Yeast(0.626) More Information | Similar Motifs Found
9	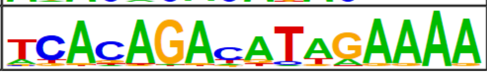	1e-56	-1.296e+02	0.19%	0.00%	27.8bp (0.0bp)	br-Z2/dmmpmm(Bergman)/fly(0.634) More Information | Similar Motifs Found

Supplementary Figure S13. HyperChIPable regions by genomic category. Pie chart displays annotations assigned with HOMER and annotation statistics calculated with HOMER for regions that are enriched by all ChIP-seq target antibodies. These regions are non-specifically enriched.

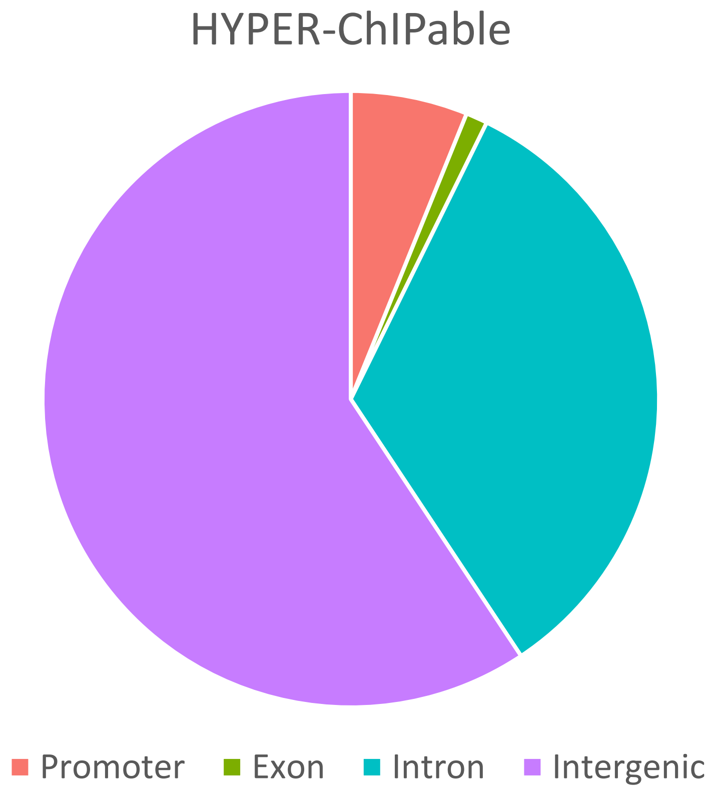

Annotation	Number of peaks	Total size (bp)	Log2 Ratio (obs/exp)	LogP enrichment (+values depleted)
TTS	5	30987562	0.025	-0.607
Exon	5	60773693	-0.946	2.526
Intron	147	1008698332	-0.121	2.211
Intergenic	278	1671437591	0.069	-2.181
Promoter	10	35083720	0.846	-2.884

Supplementary Figure S14. Detail of ChIP-seq signal in the region of key genes. (A) Close up detail of the promoter feature from Figure 4A for *ACTB* shows that the resolution is high, identifying a 700 bp region with near basepair resolution at the first intron of the gene that controls expression. **(B)** *MYADM* is an example of a gene that may have an unannotated alternative transcription start site to the far left with strong signal at H3K4me3 and H3K27ac. The Y-axis is ChIP-seq signal displayed in RPGC with input signal subtracted. Signal shows periodicity of approximately nucleosome length (~150-160 bp) this pattern is also seen in **(C)** cross-correlation plots highlighted with blue arrows.

(A) Housekeeping gene, *ACTB*

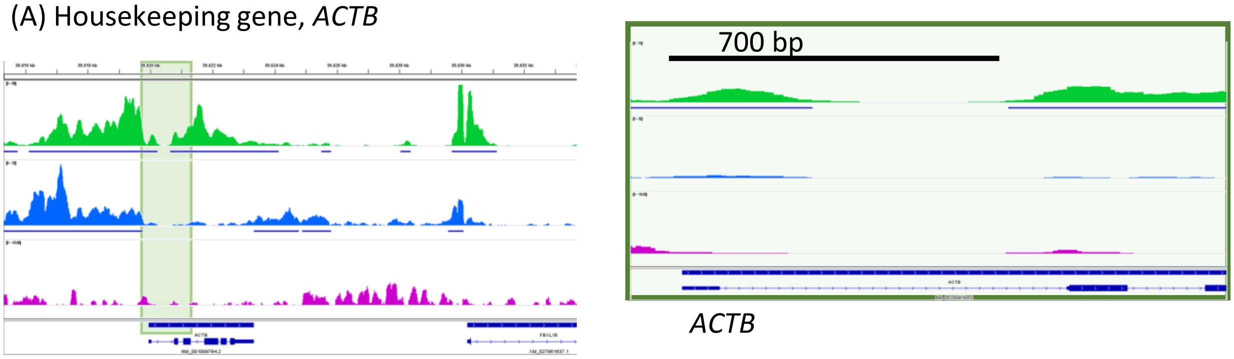

B

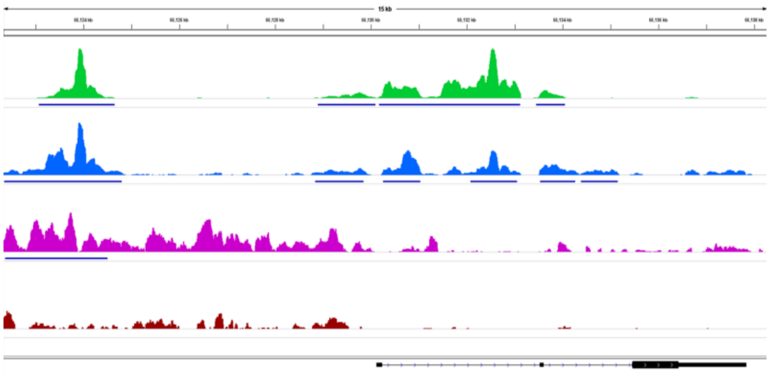

C

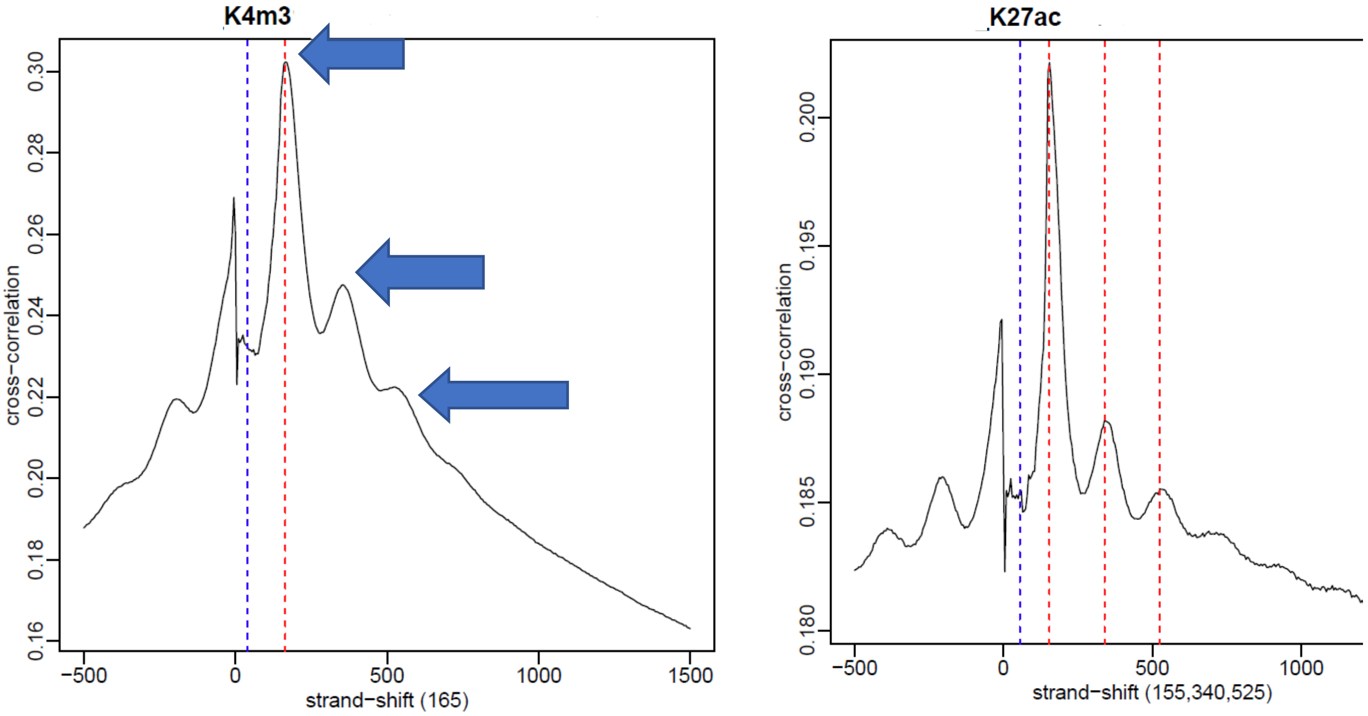

Supplement: Supplementary file 1 [file Data_Sheet_1.ZIP › New Figures and supp for re submission/Supplementary Data S1-S14 Text and Figures.pdf]
